# Modulating the evolutionary trajectory of tolerance using antibiotics with different metabolic dependencies

**DOI:** 10.1038/s41467-022-30272-0

**Published:** 2022-05-09

**Authors:** Erica J. Zheng, Ian W. Andrews, Alexandra T. Grote, Abigail L. Manson, Miguel A. Alcantar, Ashlee M. Earl, James J. Collins

**Affiliations:** 1 Program in Chemical Biology, Harvard University, Cambridge, MA 02138 USA; 2 Infectious Disease and Microbiome Program, Broad Institute of MIT and Harvard, Cambridge, MA 02142 USA; 3 Institute for Medical Engineering & Science, Department of Biological Engineering, and Synthetic Biology Center, Massachusetts Institute of Technology, Cambridge, MA 02139 USA; 4 Wyss Institute for Biologically Inspired Engineering, Harvard University, Boston, MA 02115 USA; 5 Harvard-MIT Program in Health Sciences and Technology, Cambridge, MA 02139 USA

**Keywords:** Antibiotics, Bacterial evolution, Antimicrobial resistance

## Abstract

Antibiotic tolerance, or the ability of bacteria to survive antibiotic treatment in the absence of genetic resistance, has been linked to chronic and recurrent infections. Tolerant cells are often characterized by a low metabolic state, against which most clinically used antibiotics are ineffective. Here, we show that tolerance readily evolves against antibiotics that are strongly dependent on bacterial metabolism, but does not arise against antibiotics whose efficacy is only minimally affected by metabolic state. We identify a mechanism of tolerance evolution in *E. coli* involving deletion of the sodium-proton antiporter gene *nhaA*, which results in downregulated metabolism and upregulated stress responses. Additionally, we find that cycling of antibiotics with different metabolic dependencies interrupts evolution of tolerance in vitro, increasing the lifetime of treatment efficacy. Our work highlights the potential for limiting the occurrence and extent of tolerance by accounting for antibiotic dependencies on bacterial metabolism.

## Abstract (web summary)

Antibiotic tolerance, or the ability of bacteria to survive antibiotic treatment in the absence of genetic resistance, often involves a low metabolic state. Here, Zheng et al. show that tolerance does not readily evolve against antibiotics whose efficacy is only minimally affected by bacterial metabolism, and find that cycling of antibiotics with different metabolic dependencies interrupts evolution of tolerance.

## Introduction

Antibiotic tolerance evolves in response to repeated antibiotic exposure at an alarming pace, with increases in bacterial survival of several logs observed within 3-10 days of antibiotic treatment through heritable, tolerance-encoding mutations^1–6^. These in vitro findings have been supported by several recent studies documenting the evolution of bacterial tolerance in the clinic, through longitudinal phenotyping and whole-genome sequencing^7–11^. Strategies to delay or prevent tolerance evolution and extend the lifetime of antibiotic efficacy are urgently needed^7,10,12^.

To date, studies of tolerance evolution have primarily been conducted with antibiotics that have a strong dependence on bacterial metabolism^1,3,4,13–15^, which fail to eradicate bacteria in infection-relevant contexts where metabolism can be downregulated^15–23^. Recently, we developed a metric for quantifying antibiotic metabolism dependence^15^ and found that structurally diverse antibiotics are strongly (SDM) or weakly dependent on metabolism (WDM), where WDM antibiotics retain efficacy even against dormant cells. Even though tolerance and bacterial metabolism are inherently linked^16,24^, the effect of antibiotic metabolism dependence on tolerance evolution has not been investigated, and it is unknown whether tolerance evolves similarly to all antibiotics, whether SDM or WDM. However, as metabolic dormancy protects against antibiotic lethality^16,25,26^ and in vitro antibiotic evolution experiments have yielded tolerance-inducing mutations in metabolic genes^4,27,28^, there is a strong likelihood that antibiotic metabolism dependence will be a key determinant of the rate at which tolerance evolves.

Here, we sought to determine how the bacterial evolutionary trajectory of tolerance differs during treatment with antibiotics that have different metabolic dependencies. We show that the evolvability of tolerance is largely determined by antibiotic metabolism dependence, with SDM antibiotics more readily evolving tolerance than WDM antibiotics. We discover that deletion of the sodium/proton antiporter *nhaA* during repeated SDM treatment confers tolerance through metabolic suppression. Additionally, we demonstrate that cycling of antibiotics with different metabolic dependencies delays the evolution of tolerance while reducing the use of toxic antibiotics. These results underscore the importance of considering antibiotic metabolism dependence when designing treatments that are more robust against bacterial counterstrategies.

## Results

### Tolerance evolves during repeated SDM but not WDM antibiotic exposure

**Fig. 1:**
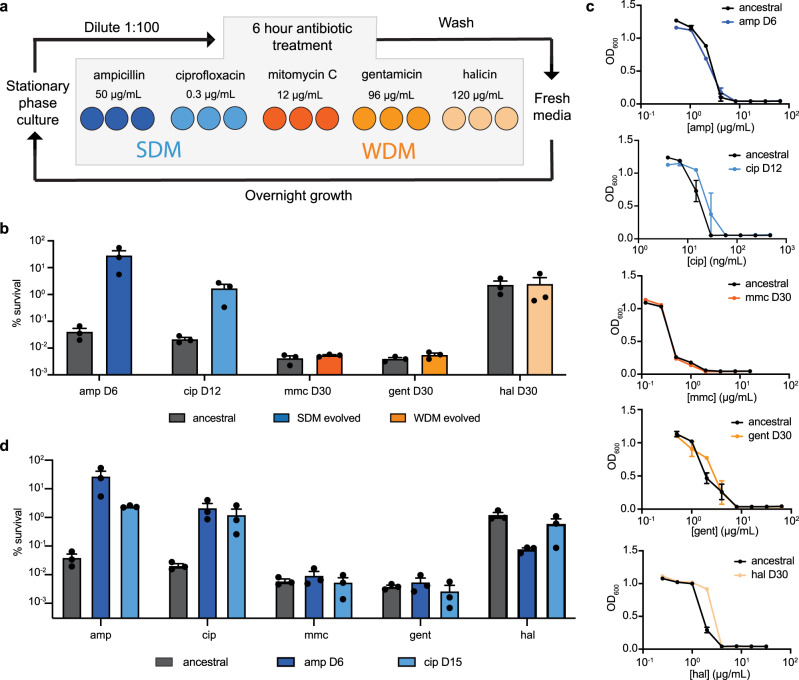
Tolerance evolves during repeated SDM but not WDM antibiotic treatment. **a** Schematic of evolution. An overnight culture of *E. coli* BW25113 was diluted 1:100 in LB, then incubated with an SDM or WDM antibiotic at the indicated concentration for 6 hours in a deep 96-well plate in triplicate. Next, plates were centrifuged and washed two times in PBS. Cells were then re-suspended in fresh LB, grown overnight, and the process was repeated the following day. **b** Percent survival of ancestral and evolved cells against SDM and WDM antibiotics. Horizontal axis labels indicate the antibiotic on which the culture was evolved and the evolution day (i.e., amp D6 indicates cells were evolved on ampicillin for 6 days). Data are representative of three biological replicates; error bars show SEM. **c** MICs of ancestral and evolved cultures. Experiments were performed in biological triplicate; error bars indicate SEM. **d** Cross-tolerance of ampicillin- and ciprofloxacin-evolved cultures. Horizontal axis labels indicate the antibiotic tested for cross-tolerance. The figure legend indicates the antibiotic on which the culture was evolved and the evolution day. Shown is the mean of three biological replicates; error bars indicate SEM. Source data are provided as a Source Data file.

To compare evolution of tolerance during SDM versus WDM antibiotic treatment, we conducted parallel antibiotic evolutions of *E. coli* BW25113 against two SDM antibiotics (ampicillin and ciprofloxacin) and three WDM antibiotics (gentamicin, halicin, and mitomycin C)^15,29^ (Fig. 1a, Supplementary Fig. 1, Supplementary Table 2). We hypothesized that over repeated SDM treatment, tolerant mutants would be continually selected for, increasing their occurrence in the population. Additionally, we hypothesized that metabolic dormancy would not offer a survival advantage during WDM treatment^15^, and thus tolerant cells would not be selected for and their proportion in the population would not increase. To test these hypotheses experimentally, we diluted overnight cultures 1:100 in fresh LB media and subjected cells to a 6-hour antibiotic treatment in biological triplicate. Cells were then washed twice in phosphate-buffered saline, re-suspended in LB, and grown overnight. This process was repeated every day over the course of the experiments.

Consistent with previous studies, ampicillin-treated cultures quickly evolved tolerance, as by day 6 we observed a 3-log increase in survival compared to the ancestral strain treated with the same concentration of antibiotic (Fig. 1b, Supplementary Fig. 3a)^1^. Cultures evolved on ciprofloxacin also developed tolerance (Fig. 1b), though several days later than ampicillin (Supplementary Fig. 3b), possibly since ciprofloxacin is less dependent on metabolism than ampicillin^15^. The increased antibiotic survival of these evolved cultures was population-wide, indicating tolerance, rather than persistence which is characterized by a biphasic kill curve (Supplementary Fig. 2b). The respective MICs remained consistent for each of these experiments, ruling out the possibility of resistance accounting for the increase in survival (Fig. 1c), and there was no evidence of heteroresistance, where a portion of the population has decreased antibiotic susceptibility^30–32^ (Supplementary Fig. 2c, d). In contrast, cultures evolved on WDM antibiotics did not develop tolerance up through day 30, where there was still no increase in survival compared to the ancestral strain (Fig. 1b).

Due to differences in killing efficacy of these SDM and WDM antibiotics against the ancestral strain, we verified that these contrasting evolutionary outcomes were not due to a population bottlenecking effect. As mitomycin C and gentamicin kill an additional log of bacteria compared to ampicillin and ciprofloxacin (Fig. 1b), it is possible that differences in tolerance evolution could be due to the limited mutational diversity imposed by smaller bottleneck events^27,33^. To assess the effect of bottleneck size, we conducted a modified version of the ampicillin evolution where only 10% of the surviving fraction after antibiotic treatment was carried over to the growth phase, in order to mimic the mitomycin C and gentamicin bottleneck size. Even with this smaller population bottleneck, cells still became tolerant to ampicillin by day 7 (Supplementary Fig. 3d). Together, these results demonstrate that the evolvability of tolerance can be largely accounted for by the metabolic dependence of the applied antibiotic.

### SDM-tolerant cells are not tolerant against WDM antibiotics

In contrast to mutations in classical resistance genes that often confer protection against a single drug class, in many cases tolerant cells have increased survival against multiple antibiotic classes^1,2,16^. We took SDM-evolved tolerant cultures and tested whether they were cross-tolerant to both SDM and WDM antibiotics. We found that ampicillin-evolved cultures were cross-tolerant to ciprofloxacin and vice versa, showing that the evolved tolerance can be non-specific to the mechanism of action of the original SDM antibiotic (Fig. 1d). However, the SDM-evolved cultures were not cross-tolerant to WDM antibiotics, consistent with the ability of WDM antibiotics to kill SDM-tolerant cells (Fig. 1d)^15^.

### Identification of a tolerance-conferring deletion through whole-genome sequencing

Previous studies have found that cells can evolve tolerance by increasing their lag time^1,3,9,11^. To examine whether our SDM-treated cells had evolved tolerance through a similar mechanism, we determined colony appearance time through time-lapsed scanning of agar plates and found that there was no change in lag time (Supplementary Fig. 4a). We also carried out growth curves and did not observe any changes in growth rate (Supplementary Fig. 4b). However, we noted that SDM-evolved cells reached a lower stationary-phase density after overnight growth compared to the ancestral strain (Supplementary Fig. 4c–e). Interestingly, the decrease in stationary-phase density in SDM-evolved cells emerged at a similar time as the evolved tolerance (Supplementary Fig. 3a, b), indicating that these phenotypes could be linked. To further explore the relationship between the reduced density and tolerance, we streaked our SDM-evolved cultures and selected clonal isolates. We found that there was some heterogeneity in the evolved cultures, and that clones that did not have increased survival under SDM antibiotic treatment also did not grow to a lower maximal density, further associating this SDM-evolved tolerance with reduced stationary-phase density (Supplementary Fig. 4f).

**Fig. 2:**
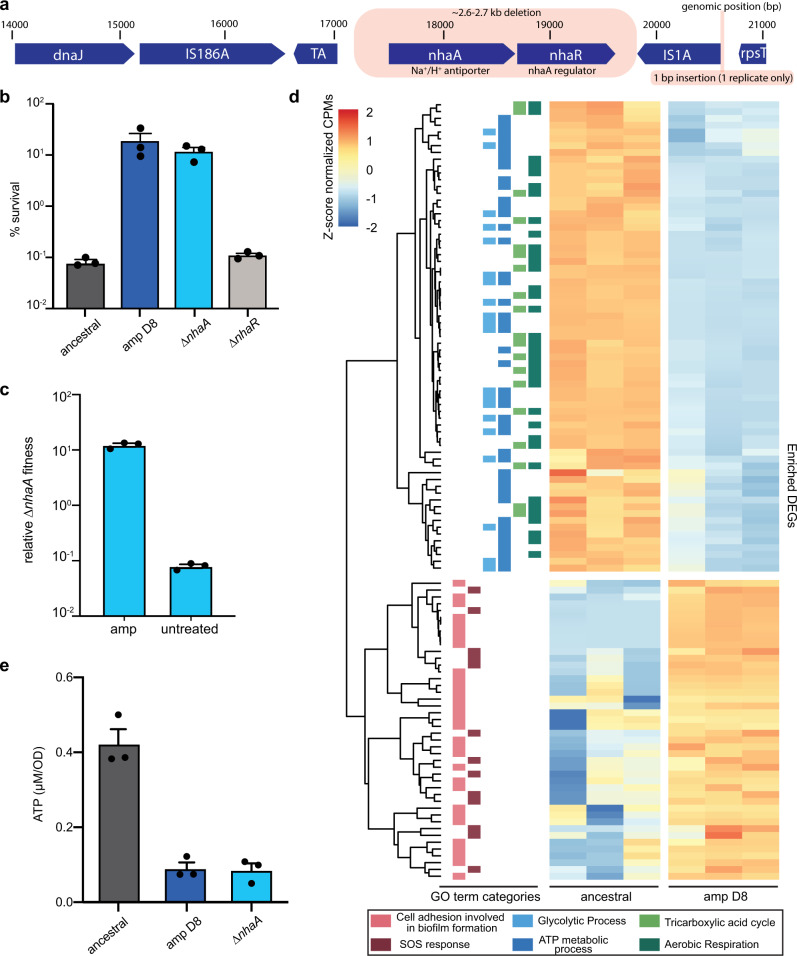
A novel mechanism of tolerance through *nhaA* loss. **a** Deletion of an ~2.6–2.7 kb region from the *E. coli* BW25113 genome, including the sodium-proton antiporter *nhaA* and its regulator *nhaR*, in all replicates of ampicillin-evolved cells was identified through whole-genome sequencing. One replicate also had a 1 bp insertion near the IS1A insertion element. **b** Percent survival of ancestral, amp D8, *ΔnhaA*, and Δ*nhaR* under ampicillin treatment. Overnight cultures were diluted 1:100 and treated with ampicillin for 6 hours. Shown is the mean of three biological replicates; error bars represent SEM. **c** Relative Δ*nhaA* fitness under ampicillin treatment and an untreated control. Overnight cultures of ancestral and Δ*nhaA* were combined and diluted 1:100 into fresh LB, then treated with ampicillin or a vehicle control (water) for 6 hours. The mean of three biological replicates is shown; error bars indicate SEM. **d** Hierarchical clustering of differentially expressed genes (DEGs) for three biological replicates of ancestral and amp D8. Heat map color shows z-score normalized counts per million (CPM). DEGs belonging to each GO term category are denoted on the left and in the bottom legend. GO term categories included are cell adhesion involved in biofilm formation (GO:0043708), SOS response (GO:0009432), glycolytic process (GO:0006096), ATP metabolic process (GO:0046034), tricarboxylic acid cycle (GO:0006099), and aerobic respiration (GO:0009060). **e** Intracellular ATP concentration in ancestral, amp D8, and *ΔnhaA*. Data are representative of three biological replicates; error bars denote SEM. Source data are provided as a Source Data file.

To identify the mechanism of the evolved tolerance, we conducted whole-genome sequencing on tolerant cultures isolated from day 8 of the ampicillin evolution (amp D8) and found ~2.6–2.7 kb deletions, which included the entirety of *nhaA*, the main sodium-proton antiporter in *E. coli*, and its regulator *nhaR* (Fig. 2a, Supplementary Table 3, Supplementary Note 1). This deletion also occurred in ciprofloxacin-evolved cells, but not in WDM-evolved cells nor in cells subjected to an untreated control evolution (Supplementary Table 4, Supplementary Fig. 5, Supplementary Note 1), indicating that this deletion appears to arise specifically under repeated SDM antibiotic treatment. We pulled the Δ*nhaA* and Δ*nhaR* single-gene knockouts from the Keio collection^34^ (Supplementary Table 1) and found that only Δ*nhaA* was SDM-tolerant (Fig. 2b, Supplementary Fig. 2). Further, in direct competition experiments with the ancestral strain, Δ*nhaA* had higher fitness under ampicillin treatment, but not under the untreated control (Fig. 2c). Overall, these results indicate that deletion of *nhaA* in SDM-evolved strains is responsible for the mutant phenotype.

### *nhaA* deletion disrupts cellular homeostasis and triggers metabolic suppression

*nhaA* is critical to the maintenance of a neutral internal pH and is highly expressed in stationary phase during growth in LB, when alkaline conditions have been reached due to catabolism of amino acids^35–38^. Interestingly, Δ*nhaA* and amp D8 grew to the same maximal density as the ancestral strain in media containing glucose, where no alkalization occurs (Supplementary Fig. 6a, b). This led us to assess whether tolerance was also dependent on overnight media alkalization or whether the physiological changes leading to SDM antibiotic tolerance arose during the treatment phase itself. We found that when *nhaA* was deleted, cells grown overnight in LB were tolerant to ampicillin regardless of the medium used for drug treatment (Supplementary Fig. 6c). However, when cells were grown overnight in conditions where there is no alkalization, no tolerance was observed even when treatment was carried out in LB (Supplementary Fig. 6c). These results suggest that loss of *nhaA* in alkaline conditions disrupts cellular homeostasis, causing SDM tolerance and a reduction in maximal density^35^.

To better understand how deletion of *nhaA* leads to antibiotic tolerance, we used RNA sequencing to compare gene expression between ancestral cells and amp D8 cells harboring the *nhaA* deletion. Amp D8 cells exhibited strong signatures of repressed metabolism, with downregulation of pathways involved in ATP metabolism, aerobic respiration, and the TCA cycle (Fig. 2d, Supplementary Note 2). Consistent with the RNA sequencing analysis, amp D8 cells had lower intracellular ATP content than the ancestral strain (Fig. 2e). The suppression of metabolism in ampicillin-evolved cells may protect from metabolism-related damage triggered by SDM antibiotics^39^. We also saw upregulation of genes involved in biofilm formation and the SOS response, both of which have been linked to antibiotic tolerance (Fig. 2d, Supplementary Note 2). Using qRT-PCR on a subset of transcripts, we verified that these trends related to metabolism, the SOS response, and biofilm formation were also present in Δ*nhaA*, and that the amp D8 and Δ*nhaA* transcriptomes appear to be highly correlated (Supplementary Fig. 8). Together, these data reveal that in response to *nhaA* deletion, ampicillin-evolved cells downregulate metabolism and upregulate stress responses, which likely contribute to the antibiotic-tolerant phenotype.

### SDM/WDM cycling delays evolution of tolerance

Previous work has shown that evolution of antibiotic tolerance can be delayed by interspersing days of antibiotic treatment with days of no treatment, and that longer intervals between treatment days increases the delay of tolerance^2^. However, prolonging the time between doses by interspersing days of treatment with no treatment may not be a viable strategy to delay tolerance in the clinic, as many clinical indications require consecutive days of antibiotic therapy^12,40,41^. Thus, we wondered if this principle could be applied to our SDM/WDM findings, where instead of alternating antibiotic treatment with no treatment, SDM antibiotic treatment could be alternated with WDM treatment in order to delay evolution of SDM tolerance while permitting daily antibiotic dosing. As WDM antibiotics eradicate cells regardless of metabolic state^15^, this strategy might stall the selection for SDM-tolerant mutants, with the benefits of minimizing the application of WDM antibiotics which often cause human toxicity^42–44^ and allowing for uninterrupted antibiotic treatment.

**Fig. 3:**
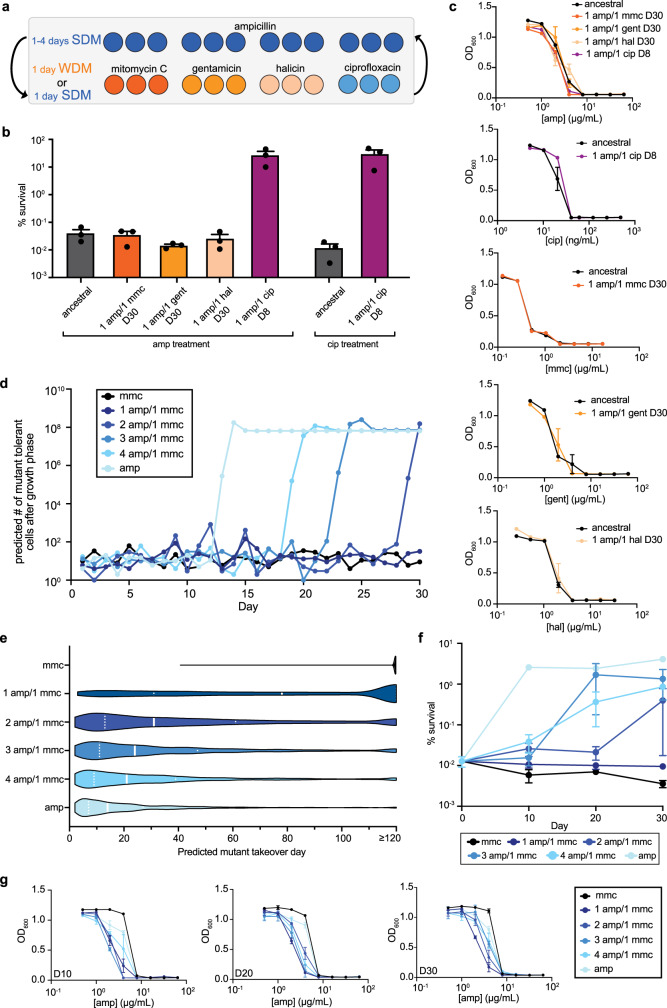
SDM/WDM cycling delays evolution of SDM tolerance. **a** Schematic of antibiotic cycling evolutions. Evolutions were conducted identically to Fig. 1, except the applied antibiotic was cycled as indicated. **b** Percent survival from evolutions where two antibiotics were alternated every day. Horizontal axis labels indicate the antibiotic(s) on which the culture was evolved and the evolution day. Survival assays were conducted with ampicillin and ciprofloxacin; survival data are grouped by the antibiotic used for phenotyping. Data are representative of three biological replicates; error bars indicate SEM. **c** MICs of evolutions where two antibiotics were alternated every day. Three biological replicates were assayed; error bars indicate SEM. **d** Predicted number of tolerant mutant cells after the growth phase of each evolution day for various ampicillin/mitomycin C cycling regimens. One thousand simulations of the model were run and the output from a simulation with the median takeover day is shown. The mutation rate parameter used for these simulations was 50 × 10^−10^ mutations per division^3^. **e** Violin plot representation of the mutant takeover day over 1000 model simulations. Mutant takeover day is defined as when the number of tolerant mutant cells exceeds the number of wild-type cells. Simulation values of 120 and greater are binned together. Solid white lines indicate the median simulation value and dotted lines represent the 25^th^ and 75^th^ percentiles. The width of each shaded area is indicative of the frequency of that value amongst the 1000 simulations, and the length of the shaded area extends from the minimum to maximum simulation value. The mutation rate parameter used for these simulations was 50 × 10^−10^ mutations per division^3^. **f** Experimental percent survival for evolutions conducted with all ampicillin, all mitomycin C, or cycling of 1–4 days of ampicillin treatment with 1 day of mitomycin C treatment. Survival assays with 50 µg/mL ampicillin were done on days 10, 20, and 30. Data are representative of three biological replicates; error bars denote SEM. **g** Ampicillin MIC for cycling regimens conducted with all ampicillin, all mitomycin C, or cycling of 1–4 days of ampicillin treatment with 1 day of mitomycin C treatment. MICs were taken from days 10, 20, and 30 of each evolution. Data are representative of three biological replicates; error bars represent SEM. Source data are provided as a Source Data file.

To first determine whether cycling of antibiotics with different metabolic dependencies can delay tolerance, we followed the same evolution protocol as with the single antibiotic (monotherapy) evolution, except every day treatment was alternated between two SDM antibiotics (ampicillin and ciprofloxacin), or between an SDM antibiotic (ampicillin) and a WDM antibiotic (mitomycin C, gentamicin, or halicin) (Fig. 3a). We observed no increase in survival to ampicillin treatment for the SDM/WDM cycling evolution up to day 30 (Fig. 3b). However, daily cycling of two SDM antibiotics (ampicillin/ciprofloxacin) quickly led to evolution of SDM-tolerance, with an increase in survival to both ampicillin and ciprofloxacin (Fig. 3b), but no change in MIC (Fig. 3c). Tolerance evolved during SDM/SDM cycling through the same mechanism as the SDM monotherapy evolutions, as we again observed deletion of *nhaA* (Supplementary Fig. 5e, Supplementary Table 4). The observation that SDM/SDM cycling was unable to prevent evolution of tolerance demonstrates that the delay in SDM tolerance evolution during cycling is dependent on combining an SDM with a WDM, rather than simply the cycling of two drugs with different mechanisms of action.

Having determined that daily SDM/WDM cycling delays evolution of SDM tolerance, we next sought to determine whether the frequency of SDM versus WDM application during cycling treatments affected the amount of tolerance delay. Reducing the frequency of WDM treatment with a concomitant increase in SDM treatment frequency could allow for more dose-sparing of the WDM antibiotic, though there would likely be a trade-off between these toxicity concerns and the amount of tolerance delay. Indeed, a stochastic evolution dynamics model predicted that shifting the balance of SDM/WDM cycling in favor of more frequent SDM treatments would cause tolerant mutants to more rapidly take over the population (Fig. 3d, e, Supplementary Note 3). However, the model also predicted that even relatively infrequent WDM dosing (e.g., up to 4 days of SDM treatment alternated with 1 day of WDM treatment) could provide some delay of tolerance evolution compared with SDM monotherapy (Fig. 3d, e). To test these predictions experimentally, we alternated 1–4 days of ampicillin (SDM) treatment with 1 day of mitomycin C (WDM) treatment, and then measured MICs and survival against ampicillin at days 10, 20, and 30 (Fig. 3f, g). Consistent with the model predictions, we found that tolerance evolved faster as the number of consecutive days of SDM treatment increased, though infrequent dosing with a WDM antibiotic still resulted in a notable delay of tolerance evolution (Fig. 3f). These results demonstrate a clear trade-off between tolerance evolution and WDM treatment frequency.

## Discussion

The relationship between antibiotic metabolism dependence and the evolution of tolerance had not been previously explored, despite low metabolism being a key contributor to antibiotic tolerance^16,20,24^. Our finding that WDM antibiotics did not evolve tolerance over the course of 30 days of treatment suggests that some antibiotics may be better suited for long-term treatment than others that are more vulnerable to tolerance evolution. While most known WDM antibiotics have limited clinical use due to toxicity^42–44^, continuing efforts in finding new compounds with anti-tolerance activity^45^, as well as the design of combination strategies that can dose-spare toxic antibiotics^15^, will be crucial for improving treatment outcomes, particularly for patients with chronic bacterial infections who often have high levels of antibiotic-tolerant isolates^46^. Indeed, a recent study found little antibiotic tolerance amongst isolates from patients with acute *P. aeruginosa* lung infections, but high levels of tolerance in patients with chronic infections^46^. Our SDM/WDM cycling work suggests that differences in antibiotic metabolic dependencies could be harnessed to guide the design of customized treatment strategies based on balancing concerns of toxicity and tolerance evolution.

While tolerance was first regarded as a physiological, non-inheritable state primarily induced by environmental cues^47^, in recent years whole-genome sequencing has shed light on the many mutations that can cause heritable tolerance. Given the size of the *E. coli* “tolerome”, genes known to contribute to tolerance^3,48–50^, it is not surprising that many different mechanisms of antibiotic tolerance evolution have been reported^5^. Here, we identify a mechanism of SDM tolerance through deletion of the sodium-proton antiporter gene *nhaA*. Despite the diverse pool of target mutations for evolved tolerance between studies, transcriptomics and proteomics have allowed for the characterization of pathways that are commonly differentially expressed across separate studies^4,5,13,14,51^. Consistent with many previously identified antibiotic-tolerant mutants, our transcriptomic analysis of SDM-tolerant mutants revealed upregulation of the SOS response and biofilm formation along with dramatic downregulation of metabolic processes^4,14,24,51,52^. Metabolic mutations have not only been implicated in tolerance, but also in resistance, as shown by recent studies which found mutations in central carbon metabolism, energy metabolism, and biosynthetic pathway genes associated with antibiotic resistance in in vitro evolved strains and in clinical isolates^28,53^. A clearer, more comprehensive picture of cellular pathways that can lead to tolerance and resistance is emerging, which will be helpful in developing more robust antibiotic treatments.

Modulation of both intracellular and extracellular pH has been known to alter antibiotic susceptibility^51,54,55^. For example, mutations in respiratory complex I were found during an in vitro evolution of *E. coli*, which resulted in cytoplasmic acidification leading to increased antibiotic persistence^51^. Antibiotic treatment itself is also thought to trigger changes in intracellular pH, which activates cell stress and contributes to antibiotic-induced cell death, though whether this is achieved through alkalization or acidification may be dependent on the species and antibiotics in question^54,56,57^. Here, we demonstrate an additional link between pH and antibiotic efficacy whereby *nhaA* deletion results in tolerance, specifically under alkaline conditions. However, it remains unclear how *nhaA* deletion triggers metabolic repression and antibiotic tolerance, and the range of biological contexts under which this result would be observed. Considering the role of *nhaA* in maintaining a neutral intracellular pH under alkaline conditions^35^, and our observation that antibiotic tolerance mediated by *nhaA* loss was dependent on alkalinity (Supplementary Fig. 6), *nhaA* deletion may not trigger this metabolic reprogramming and antibiotic tolerance if treatment were conducted under consistent neutral or acidic pH conditions^58^. Nonetheless, we expect the observations of differing tolerance evolution rates between SDM and WDM antibiotics to hold for any metabolism-altering mutations that lead to tolerance. While tolerance has in most cases been linked to a low metabolic state, there may be tolerance-conferring mutations that do not impact metabolic state, such as increased antibiotic efflux, in which case the evolutionary outcome may not be impacted by antibiotic metabolism dependence^59,60^. More work is required to understand the relationships between *nhaA* activity, metabolism, and antibiotic efficacy.

As we strive to preserve long-term antibiotic efficacy, we must consider not only resistance but tolerance as well. The evolution of tolerance has been shown to facilitate the evolution of resistance^3,5,11,46,61–65^, and thus anti-tolerance strategies should also be effective in delaying antibiotic resistance, though the interplay of tolerance and resistance evolution under combination treatments is further complicated when considering drug pairs that interact through, for example, suppression^11^. Cycling strategies, where antibiotics are alternated periodically, are able to slow the evolution of resistance when employing pairs of antibiotics that are collaterally sensitive, which then constrains the available mutational pathways to resistance against both antibiotics^66^. Here, we found that cycling strategies are also effective in delaying tolerance, but only when SDM antibiotics are cycled with WDM antibiotics, which slow the selection for SDM-tolerant mutants. Our findings emphasize the need to account for bacterial metabolism in determining antibiotic efficacy as well as the evolutionary landscape of tolerance in response to repeated antibiotic challenge.

## Methods

### Evolution protocol

An overnight culture of *E. coli* BW25113 (Supplementary Table 1) was diluted 1:100 in LB broth and added to a deep 96-well plate with antibiotic for a total volume of 400 µL. Concentrations of antibiotics used were as follows: 50 µg/mL ampicillin (amp), 0.3 µg/mL ciprofloxacin (cip), 12 µg/mL mitomycin C (mmc), 96 µg/mL gentamicin (gent), and 120 µg/mL halicin (hal). Plates were sealed with BioExcell Films and incubated at 37 °C with 900 rpm shaking for 6 hours, then centrifuged at 3000 × *g*, 4 °C for 7 min. Cells were washed twice with PBS, then re-suspended in 400 µL of fresh LB broth for 18 h of growth at 37 °C with shaking. The following morning, the OD_600_ of the overnight cultures was measured in a Spectramax M3 Plate Reader (Molecular Devices), then overnight cultures were diluted 1:100 and antibiotic was added to initiate the next evolution round. This process was repeated every day throughout the course of the evolutions. Overnight cultures from each day were saved as 25% glycerol stocks and stored at −80 °C. Each evolution condition was conducted in biological triplicate.

For monotherapy evolutions, the same antibiotic was applied daily. The untreated control evolution was conducted identically to antibiotic-treated evolutions, except a vehicle control (water) was added during the treatment phase. For SDM/SDM cycling evolutions, ampicillin, and ciprofloxacin treatment was alternated every day. For SDM/WDM cycling evolutions, 1–4 days of ampicillin treatment was alternated with 1 day of WDM treatment.

For population bottleneck analysis, cultures were evolved on ampicillin as described above, except only 10% of cells surviving antibiotic treatment were carried over into the overnight growth phase.

### Antibiotic survival assays

Overnight cultures grown from frozen stocks were diluted 1:100 in fresh LB in a deep 96-well plate. Antibiotic was added at the following concentrations: 50 µg/mL amp, 0.3 µg/mL cip, 12 µg/mL mmc, 96 µg/mL gent, and 120 µg/mL hal. Plates were incubated at 37 °C with 900 rpm shaking for 6 hours, then centrifuged at 3000 × *g*, 4 °C for 7 min, and washed twice with PBS. Cells were then re-suspended in PBS and serially diluted 10-fold. 7 µL was spotted on LB agar plates, which were then incubated overnight at 37 °C for colony enumeration the following day. All survival assays were performed on the population level from frozen stocks saved from overnight cultures of each evolution day, except for the clonal analysis in Supplementary Fig. 4f, where frozen stocks of overnights saved from the indicated evolution day were streaked on LB agar plates, then single colonies were inoculated into LB media and grown overnight for survival and optical density analyses.

For media comparison assays (Supplementary Fig. 6), cells were grown overnight in LB, MOPS minimal media (Teknova M2106) with 0.2% glucose, or LB supplemented with 0.2% glucose. These overnight cultures were then washed and diluted 1:100 in fresh media as indicated for antibiotic survival assays.

### Determination of MIC

Overnight cultures were diluted 1 in 10,000 in fresh LB (except for Supplementary Fig. 2c, where overnight cultures were diluted 1 in 100) and distributed into 96-well round-bottom clear polypropylene plates (Corning) with two-fold dilutions of antibiotic for a total volume of 100 µL. Plates were sealed with AeraSeal membranes (Sigma-Aldrich) and incubated for 24 hours at 37 °C, 900 rpm shaking, then read at OD_600_ in a Spectramax M3 plate reader.

### Population analysis profiling

Population analysis profiling was used to determine presence or absence of heteroresistance as described by Andersson et al.^30^. Overnight cultures were serially diluted 10-fold and spread on LB agar plates containing ampicillin at the indicated concentrations. Plates were incubated at 37 °C and colonies were counted the following day. Heteroresistance was determined as the presence of a subpopulation at a frequency of 10^−7^ or greater with a fold increase in resistance of at least 8-fold or more as compared to the main population.

### Growth rate determination

Overnight cultures were diluted 1:100 in fresh LB and 100 µL was added to 96-well round-bottom clear polypropylene plates (Corning). To prevent evaporation, 35 µL of mineral oil was added to the liquid surface. Growth curves were read in a Spectramax M3 plate reader, at 37 °C with shaking at an optical density of 600 nm; reads were taken every 5 min. To determine growth rate, the log phase of growth curves was fit to an exponential growth equation using Graphpad Prism 8.

### Colony appearance time determination

Overnight cultures were diluted by a factor of 10^5^–10^6^, and 75 µL was spread on a round LB agar plate to achieve 100–300 cells per plate. Plates were placed agar side down on an Epson translucent-imaging scanner at 37 °C and scans were taken every 5 min. ColTapp^67^ was used to determine colony appearance time.

### Whole-genome sequencing

The ancestral *E. coli* BW25113 and two replicate wells of amp D8 were sent for bacterial whole-genome sequencing. Briefly, overnight cultures were grown from frozen stocks in LB broth and 1 mL was pelleted. Cell pellets were immediately frozen at −80 °C and sent to Quintara Biosciences (Cambridge, MA) for DNA extraction and sequencing. DNA was extracted using the Universal Genomic DNA Kit (CW Biosciences), then sequenced on a NovaSeq S4 flow cell, with 2 × 150 paired-end reads at 100X coverage. BWA (v0.7.17)^68^ was used to align sequencing reads to the *E. coli* BW25113 reference genome (NCBI CP009273.1), then Pilon (v1.23)^69^ with default settings was used to identify variants. We verified that short variants had mapping quality >10 and filtered large indels and duplications designated as “imprecise” by Pilon (containing N’s in the local reassembly), leaving just three variants (Supplementary Table 3).

### Verification of deletion

Frozen stocks were streaked on LB agar and single colonies were picked into 250 µL sterile water and vortexed. Colony PCR reactions were set up using Q5 High-Fidelity 2X Master Mix (New England Biosciences) containing 1 µL of re-suspended cells. For primer set 1, an annealing temperature of 61 °C and 2.5-min extension time was used. For primer set 2, the annealing temperature was 61 °C with a 4-min extension time. Primer set 2 probes a larger flanking region of *nhaA/nhaR* and was used to identify the deletion in ciprofloxacin-evolved and ampicillin/ciprofloxacin-evolved cells (Supplementary Data 1). All reactions included an initial 5-min step at 98 °C for cell lysis. PCR reactions were run on an agarose gel with a 1 kb ladder (New England Biosciences) and imaged with an Azure Biosystems c400 Imaging System. PCR products with a deletion were cleaned using the Qiagen PCR Clean-Up Kit, then sent to Quintara Biosciences (Cambridge, MA) for Sanger sequencing. Geneious (Biomatters) was used to map Sanger sequencing results onto the ancestral genome (NCBI CP009273.1).

### pH measurements

Extracellular pH was measured using a Mettler Toledo FiveEasy pH meter. The pH meter was calibrated according to the manufacturer’s instructions, then used to measure the pH of the media at inoculation and after overnight growth.

### Determination of relative fitness

Stationary phase ancestral and Δ*nhaA* were mixed 1:1, then diluted 1:100 into fresh LB in a deep 96-well plate. 50 μg/mL amp or a vehicle control (water) was added and plates were incubated for 6 hours at 37 °C with 900 rpm shaking. Plates were then spun down at 4 °C, 3000 × *g* for 7 min, washed twice with PBS, then re-suspended in an equal volume of PBS. Cells were serially diluted 10-fold in PBS, then 7 µL was spotted on both LB agar and LB agar containing 50 µg/mL kanamycin. Agar plates were incubated at 37 °C overnight then counted for colony enumeration. The number of Δ*nhaA* cells was taken as the colony counts on LB agar containing kanamycin, and the total number of cells (Δ*nhaA* and ancestral) was taken as the colony counts on LB agar containing no antibiotic.

Relative fitness was calculated according to Van den Bergh et al.^2^. Proportion composition of Δ*nhaA* was determined by dividing the Δ*nhaA* CFU/mL by the total CFU/mL prior to antibiotic treatment and after treatment. Fitness (*F*) of Δ*nhaA* relative to the ancestral strain was then calculated as

1 $$F=\frac{p\left(T\right)-[p\left(T\right)* p\left(0\right)]}{p\left(0\right)-[p\left(T\right)* p\left(0\right)]}$$

where *p(T*) is the proportion composition of Δ*nhaA* after the 6 hour treatment period and *p(0*) is the proportion composition of Δ*nhaA* immediately prior to treatment^2^.

### RNA sequencing and analysis

Overnight cultures grown in LB of the ancestral strain and amp D8 cultures were prepared in biological triplicate, then pelleted. Cell pellets were frozen at −80 °C and sent to Quintara Biosciences (Cambridge, MA) for RNA extraction and sequencing. RNA was extracted using the RNeasy Mini Kit (Qiagen), then sequenced on a NovaSeq S4 flow cell, with 2 × 150 paired-end reads at 100X coverage.

Quality of sequencing reads from each sample was assessed using FastQC (11.9)^70^. Reads were aligned to the *E. coli* BW25113 complete genome, CP009273.1, using Bowtie2 (2.3.4.3)^71^, and read counts were assigned to genes using HTSeq (2.0.1)^72^. Read counts were used as input to EdgeR (Version 3.32.1) to determine differentially expressed genes between the evolved group and the untreated control using default settings^73,74^. Because EdgeR suggests filtering of genes with very low counts across all libraries, we used an expression cutoff of at least 5 CPMs (Counts Per Million), calculated using EdgeR, in at least two biological replicates, consistent with EdgeR recommendations. Genes were determined to be differentially expressed using standard EdgeR settings, *p* < 0.05 and false-discovery rate (FDR) of < 0.05. CPMs were used for the unsupervised clustering and heatmap generation. GO term enrichment on Biological Process GO terms was performed using Fisher’s exact test implemented in OmicsBox (2.0.36)^75^.

Gene Set Enrichment Analysis (GSEA) was performed using OmicsBox with default settings (1000 permutations and a classic enrichment statistic, min gene set = 15, max gene set = 500) and an FDR of < 0.05. A ranked list of expressed genes was generated using log Fold Change. GSEA was used to determine which gene sets were enriched at either the top or the bottom extreme of the ranked list of genes.

### qRT-PCR

RNA extraction was performed as described in Culviner et al.^76^. Briefly, 1 mL TRI Reagent RT (Molecular Research Center) was added to cell pellets from 1 mL of overnight cultures and incubated in a 70 °C bead bath for 10 min. Tubes were frozen at −80 °C for 15 min, then thawed at room temperature and spun in a temperature-controlled microfuge at 4 °C, 13,000 × *g* for 5 min. The supernatant was then transferred to a separate tube and RNA isolation was performed according to the TRI Reagent RT method (Molecular Research Center). 50 μL bromoanisole was added to the supernatant, samples were vortexed and incubated on ice for 5 min, then spun at 13,000 × *g* for 15 min at 4 °C. 400 μL of the top aqueous phase layer was transferred to a new tube and an equal volume of isopropanol was added. The mixture was then loaded on a silica column and washed once with Buffer RW1 (Qiagen) and twice with Buffer RPE (Qiagen). RNA was then eluted from the column using water, and a DNase treatment was performed for 40 min at 37 °C. Finally, RNA was re-loaded on a silica column, column washes were conducted as before, and RNA was eluted.

qRT-PCR reactions were performed using the Luna Universal One-Step RT-qPCR kit (New England Biolabs) and a LightCycler 96 (Roche). Reactions were run in technical triplicate and biological duplicate in white LightCycler 480 96-well plates (Roche). qRT-PCR primers are described in Supplementary Data 1. Threshold cycle (Ct) values were determined using the LightCycler Software (Roche).

The ΔΔCt method^77^ was used to determine relative gene expression, where first the ΔCt for each strain and each experimental gene was calculated as

2 $$\,\varDelta Ct=Ct(experimental\,gene)-Ct(housekeeping\,gene)$$

Next, the ΔΔCt for each experimental gene in either amp D8 or Δ*nhaA* was calculated as

3 $$\varDelta \varDelta Ct=\varDelta Ct(amp\,D8\,or\,\varDelta nhaA)-\varDelta Ct(ancestral)$$

The negative ΔΔCt was taken as the relative gene expression (Supplementary Fig. 8), such that a positive value indicates upregulation and a negative value indicates downregulation. Five different housekeeping genes were selected from non-differentially expressed genes as determined in the RNA sequencing analysis between amp D8 and ancestral, and each housekeeping gene was used to generate a separate ΔΔCt value for each experimental gene in order to control for any differences in these non-differentially expressed genes.

### Measurement of intracellular ATP

Intracellular ATP was measured using the BacTiter-Glo Microbial Cell Viability Assay (Promega G8230), according to the manufacturer’s instructions. Briefly, overnight cultures were diluted 1:100 and OD_600_ was measured. Diluted cells were then mixed 1:1 with the BacTiter-Glo reagent in a white 96-well polystyrene plate in technical triplicate and luminescence was measured using a Spectramax M3 plate reader. Ten-fold dilutions of ATP were used to create a standard curve.

### Determination of metabolism dependence

Metabolism dependence values (Supplementary Fig. 1c) were determined as described previously^15^. Briefly, an overnight culture of *E. coli* BW25113 was grown in LB, then diluted 1 in 10,000 and sub-cultured for 4 hours. Cells were centrifuged at 4000 × *g*, 4 °C for 15 min, washed three times with PBS, then re-suspended in 0, 0.1, 1, 10, or 100% LB in PBS. After 2 hours incubation at 37 °C, cells were back-diluted in the same nutrient conditions to an OD_600_ of 0.01 (~10^6^ CFU/mL) and subjected to halicin treatment for 3 hours. Cells were then washed twice with PBS and serially diluted for cell counting on LB agar plates. Survival under varying nutrient conditions was plotted against intracellular ATP (as determined in Zheng et al.^15^) for halicin concentrations of 2X MIC and above; linear regression was then performed and the metabolism dependence value was taken as the negative slope^15^. Linear regression was performed using Graphpad Prism 8, and a summary of the linear regression statistics is provided in Supplementary Table 2.

### Stochastic evolution dynamics model

We built a simple Monte Carlo stochastic evolution dynamics model in order to simulate the impact of different cycling regimens on tolerance evolution. The model is composed of three distinct phases: an initialization phase followed by alternating growth and treatment phases. The model was built using Matlab (version R2021a).

#### Initialization phase

Values from the experimental set-up, experimental data, and the literature are used to parameterize the model (Supplementary Table 5). A distinct set of stationary-phase density and survival percent values are randomly generated for each simulation from a uniform distribution bounded by experimentally observed values (Supplementary Table 5). Stationary phase densities are converted to stationary-phase cell numbers such that the model runs on integer values of numbers of wild-type and mutant bacteria.

#### Growth phase

The growth phase takes in numbers of wild-type and mutant cells, wild-type and mutant stationary-phase cell numbers, and the mutation rate. It returns the number of wild-type and mutant cells at the end of the growth phase of the cycle. Wild-type and mutant cells are doubled until reaching the final stationary-phase cell number. At each doubling, wild-type cells are given an opportunity to mutate to a tolerant mutant by sampling a random number from a Poisson distribution with a mean computed by multiplying the number of wild-type cells by the mutation rate. This phase makes a few simplifying assumptions. First, it assumes that all growth properties are the same between wild-type and mutant bacteria besides final stationary phase cell number. Second, the final stationary-phase cell number at the end of the growth phase, relative to the input wild-type and mutant stationary-phase cell numbers, is assumed to be linearly proportional to the number of input wild-type and mutant cells. Third, it assumes that mutants do not revert.

#### Treatment phase

The treatment phase takes in numbers of wild-type and mutant cells, wild-type and mutant survival percentages for the relevant SDM or WDM drug treatment, and the fold dilution before treatment; it returns the number of surviving wild-type and mutant cells. First, wild-type and mutant cells are diluted by sampling random numbers from a Poisson distribution with a mean computed by dividing the number of wild-type and mutant cells by the fold dilution. The surviving number of wild-type and mutant cells is then calculated by sampling random numbers from a Poisson distribution with a mean computed by multiplying the relevant survival fraction by the number of wild-type or mutant cells.

#### Analysis

Mutant takeover day is defined as the day when the mutant cell number is greater than the wild-type cell number after the growth phase. The probability of mutant takeover by a specified day was determined by computing the empirical cumulative density function for the distribution of mutant takeover days. Probabilities for days not represented in the empirical cumulative density function were estimated using linear interpolation.

### Reporting summary

Further information on research design is available in the Nature Research Reporting Summary linked to this article.

## Supplementary information

Supplementary Information

Reporting Summary

Description of Additional Supplementary Files

Supplementary Software

Supplementary Data 1

Supplementary Data 2

## Data Availability

The sequencing data generated in this study have been deposited in the Sequence Read Archive repository under accession code PRJNA810430. The reference *E. coli* BW25113 genome used in this study is available in the NCBI database under accession code CP009273.1. Source data are provided with this paper.

## Source data

Source Data

## Code availability

Code for the SDM/WDM cycling prediction model is available as Supplementary Software.
