# Supplementary material for: Modulating the evolutionary trajectory of tolerance using antibiotics with different metabolic dependencies
Source: Nat Commun. 2022 May 9;13:2525. doi: 10.1038/s41467-022-30272-0 (PMC9085803; doi:10.1038/s41467-022-30272-0)
Supplement: Supplementary file 1 — Supplementary Information [file 41467_2022_30272_MOESM1_ESM.pdf]

Supplementary Note 1: Identification of *nhaA* deletion

We sent one ancestral sample and two biological replicates of ampicillin Day 8 (amp D8) cultures for whole-genome sequencing, resulting in the identification of the *nhaA* and *nhaR* deletions in both amp D8 replicates, along with a 1 bp insertion close to a nearby IS1A insertion element in one amp D8 replicate, which were the only variants that met our filters (Fig. 2a, Supplementary Table 3). NhaA is known to play a key role in the cellular response to alkali stress and when deleted, cultures have reduced stationary-phase survival in alkaline conditions[1,2]. Transcriptional regulation of *nhaA* is growth-phase dependent: in log phase, NhaR regulates *nhaA* transcription, while in stationary phase RpoS, the stationary-phase stress-response sigma factor, solely regulates *nhaA* transcription[1].

Using colony PCR, we verified that this deletion removing *nhaA* and *nhaR* occurred in all three biological replicates of amp D8, with a consistent downstream coordinate but some variation in the upstream coordinate (Supplementary Fig. 5a-b, Supplementary Table 4). As ciprofloxacin-evolved cells had a similar phenotype to cells evolved on ampicillin, we also tested whether they harbored deletions in this region (Supplementary Data 1). This revealed a consistent deletion of 3.9 kb that removed *nhaA*, *nhaR*, the upstream toxin/antitoxin genes, and a portion of the IS186A insertion element (Supplementary Table 4, Supplementary Fig. 5c-d). We determined that deletion of *nhaA* was responsible for the mutant phenotype, as Δ*nhaA* was SDM-tolerant (Fig. 2b, Supplementary Fig. 2), grew to a reduced stationary-phase density (Supplementary Fig. 4e, g), and exhibited no change in lag time (Supplementary Fig. 4a), consistent with SDM-evolved

cultures, whereas the Δ*nhaR* phenotype was indistinguishable from the ancestral strain (Fig. 2b, Supplementary Fig. 4g).

The mechanism of this deletion is likely related to the insertion elements that flank *nhaA* and *nhaR* on either side. These types of elements, along with recombination, can cause genomic changes such as large deletions, inversions, and duplications[3]. In addition, changes involving these elements can be imprecise, as seen by the slightly different deletion boundaries amongst replicates[3].

Supplementary Note 2: Transcriptomics on SDM-tolerant cells reveal signatures of repressed metabolism and disrupted cellular homeostasis

Since the tolerance phenotype is dependent on alkalinity, we hypothesized that loss of *nhaA* disrupts regulation of internal pH, priming defense mechanisms and leading to tolerance. Indeed, previous work has shown that pre-treatment with stressors such as hydrogen peroxide activates stress pathways, shuts down metabolism, and increases antibiotic survival[4-7].

To assess the state of the cell immediately prior to antibiotic treatment, we used RNA sequencing to compare gene expression between ancestral and ampicillin-evolved cells after overnight growth in LB. We sent three biological replicates of ancestral and amp D8 cultures for RNA sequencing and observed a marked transcriptional shift in amp D8, with 1877 differentially expressed genes (DEGs) (903 upregulated, 974 downregulated) (Supplementary Data 2). Among downregulated genes, 26 out of the top 30 most significantly enriched downregulated GO terms

(FDR<0.05, Fisher's exact test) were all associated with metabolism, including aerobic respiration, ATP metabolic process, and generation of precursor metabolites and energy (Supplementary Data 2, Fig. 2d). Iron-sulfur cluster assembly, the suppression of which is known to protect against antibiotic lethality[8], also surfaced as a significantly enriched downregulated GO term (Supplementary Fig. 7a). Among upregulated genes, the most significantly enriched GO terms were related to ribosome assembly and biogenesis; however, the majority of ribosome-associated transcripts had only modest changes in gene expression (Supplementary Data 2). As such, using Gene Set Enrichment Analysis (GSEA), which takes into account the fold change in expression, no ribosome-related GO terms were found to be significantly enriched (FDR<0.05) among the upregulated genes (Supplementary Data 2).

Although they did not meet our most stringent cutoff (FDR<0.05), additional key GO terms were enriched among upregulated genes using a looser threshold (p-value <0.01; Fisher's exact test), including the SOS response, which is known to be involved in the alkali stress response[9,10] and antibiotic tolerance[11,12], as well as cell adhesion involved in biofilm formation (Fig. 2d). Interestingly, in the GSEA analysis the top three most significantly enriched upregulated terms (FDR <0.05) were all related to biofilm formation and adhesion (Supplementary Data 2), which has been linked to repressed metabolism and reduced antibiotic efficacy[13,14].

We also examined the expression of individual genes related to the role of *nhaA* in maintaining cellular homeostasis. Alternate cation-proton antiporters (Supplementary Fig. 7b), as well as transcripts associated with the osmotic stress response (Supplementary Fig. 7c), were

upregulated, consistent with the function of *nhaA* in maintaining internal pH and osmolarity[15,16]. In fact, the cation-proton antiporter *chaA* was the most significantly upregulated gene (Supplementary Data 2). Overall, the RNA sequencing results demonstrate that cells lacking *nhaA* have suppressed metabolism and disrupted homeostasis, which are often associated with antibiotic tolerance[5,8,13,14,17].

Supplementary Note 3: Sensitivity of model predictions to mutation rate parameter

We note that the quantitative predictions from our SDM/WDM cycling model are sensitive to parameters such as the mutation rate; nevertheless, sensitivity analysis of the mutation rate reveals that the same qualitative relationship between different WDM dosing frequencies holds across a range of mutation rates that might be seen with distinct mutation types (Supplementary Fig. 9).

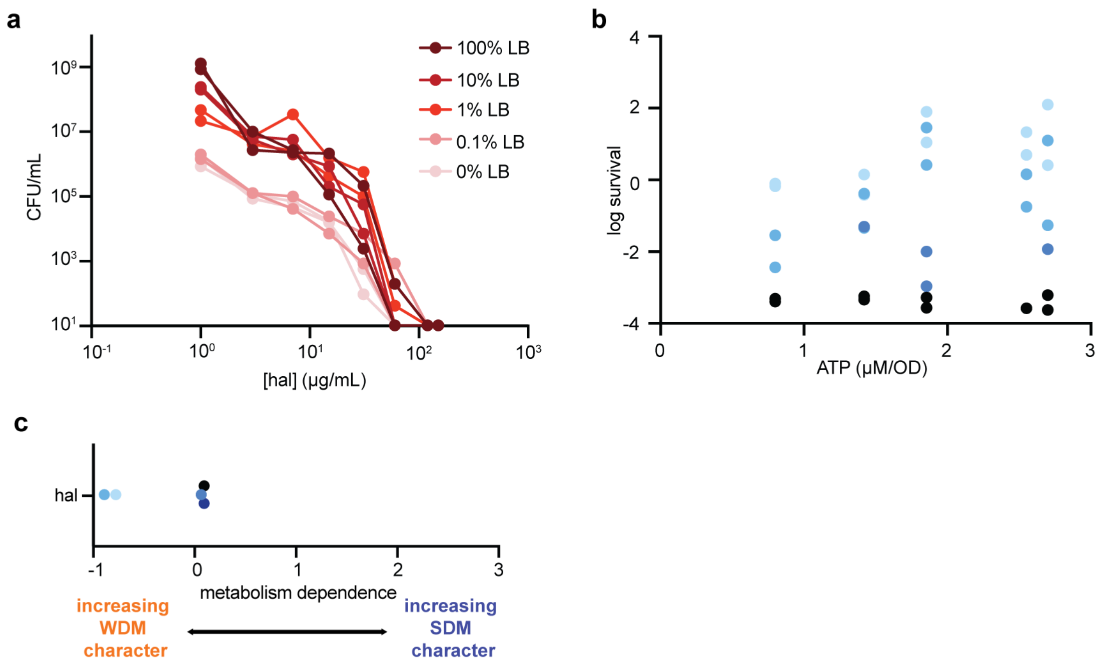

Supplementary Figure 1. Halicin is a WDM antibiotic. Related to Fig. 1 and 3. **a,** *E. coli* BW25113 was treated with halicin in 0, 0.1, 1, 10, or 100% LB in PBS for 3 hours in biological duplicate. Starting cell density was ~10^6 CFU/mL. **b,** Survival under halicin treatment as a function of intracellular ATP. Data from panel **a** at concentrations of 2X MIC or greater were included, with light to dark shading indicating increasing halicin concentration. Intracellular ATP determination is from Zheng et al[18]. Two biological replicates are shown. **c,** Metabolism dependence of halicin. Linear regression was performed on the survival vs. ATP data in panel **b** (two biological replicates) at antibiotic concentrations of 2X MIC and above, and the negative slope from this regression was taken as the metabolism dependence value. Regression data are shown in Supplementary Table 2. Light to dark shading indicates increasing halicin concentration. Source data are provided as a Source Data file.

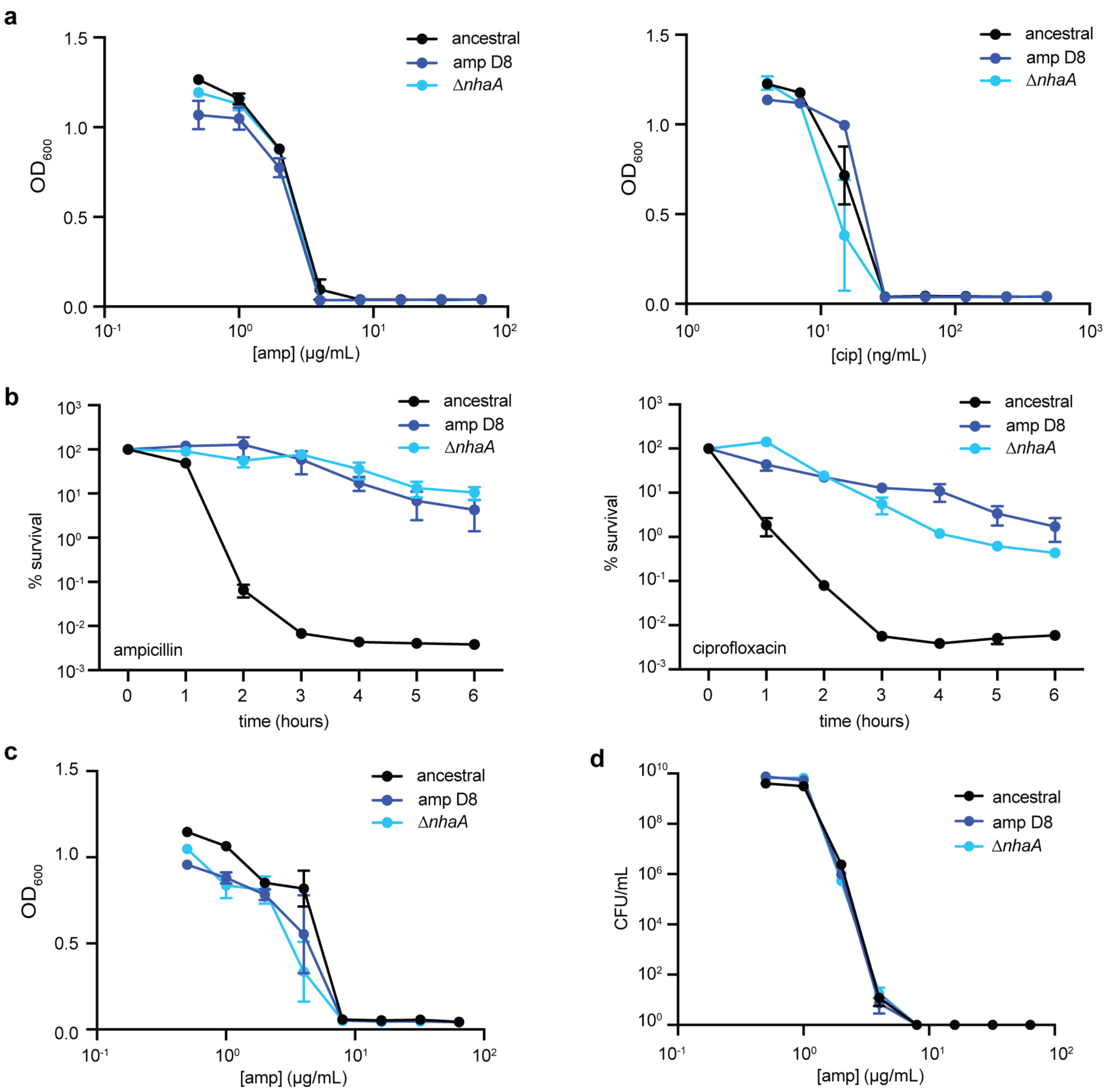

Supplementary Figure 2. Increased SDM-survival is due to antibiotic tolerance, not persistence, resistance, or heteroresistance. Related to Fig. 1 and 2. **a,** MICs were taken by diluting an overnight culture of ancestral, amp D8, or Δ*nhaA* 1 in 10,000 and adding in 2-fold dilutions of ampicillin (left) or ciprofloxacin (right). OD_{600} readings were taken after a 24-hour incubation period at 37 °C. Three biological replicates are shown; error bars denote SEM. **b,** Time-kill curves were taken by diluting an overnight culture of ancestral, amp D8, or Δ*nhaA* 1:100 and treating

with 50 µg/mL ampicillin (left) or 0.3 µg/mL ciprofloxacin (right). Aliquots were taken every hour and plated for colony forming units. Shown is the mean of three biological replicates with SEM. **c,** For detection of low-frequency heteroresistance, MICs were conducted as described in panel A, except overnight cultures were diluted 1 in 100 to generate the same inoculum as the survival assays. Data are representative of three biological replicates with SEM. **d,** Population analysis profiling where cultures were serially diluted 10-fold and plated on solid LB agar with the indicated concentration of ampicillin. Enumeration of colony-forming units was performed the following day. Shown is the mean of three biological replicates; error bars indicate SEM. Source data are provided as a Source Data file.

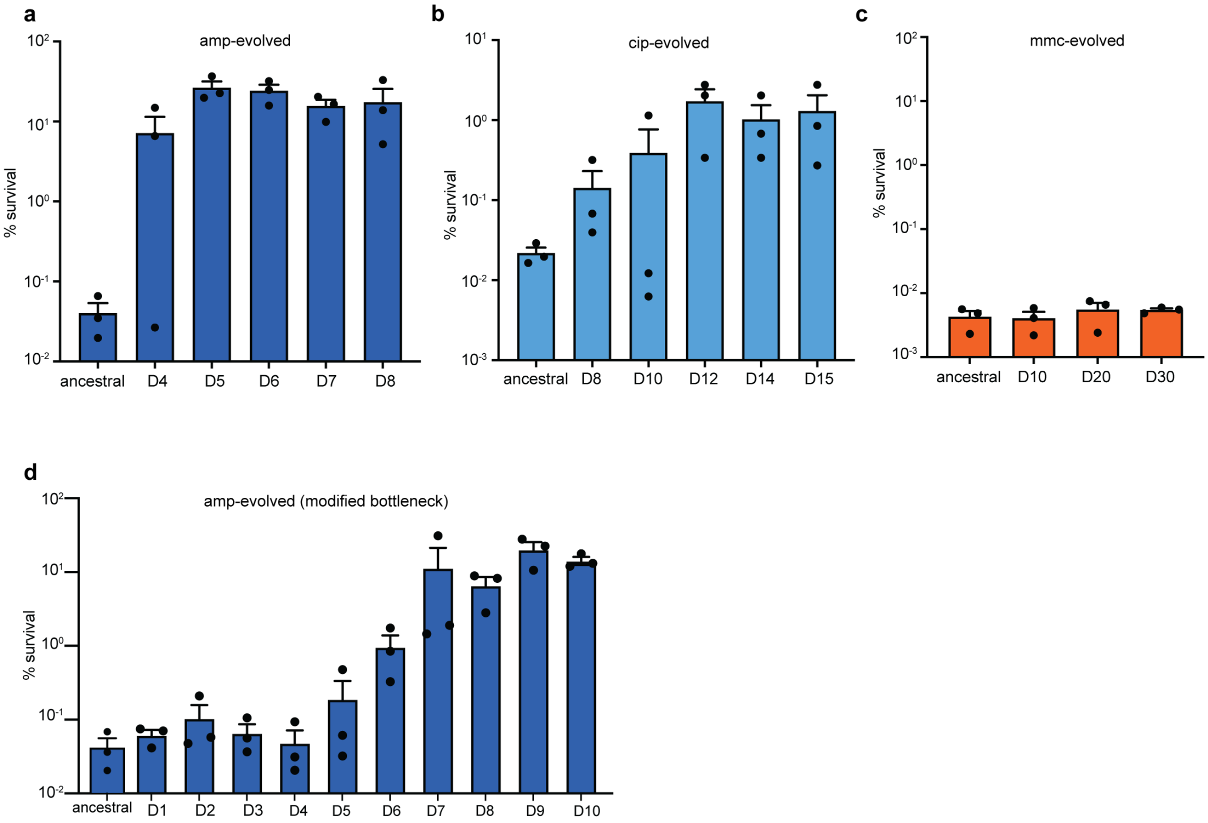

Supplementary Figure 3. Tolerance evolves at different rates for antibiotics with differing metabolic dependencies. Related to Fig. 1. **a-c,** Survival of ampicillin-evolved (a), ciprofloxacin-evolved (b), and mitomycin C-evolved (c) cultures against their respective antibiotics. Labels on the x-axis indicate the day of the evolution. Shown is the mean of three biological replicates; error bars denote SEM. **d,** A smaller population bottleneck does not delay evolution of tolerance against ampicillin. A modified ampicillin evolution was conducted where only 10% of cells surviving antibiotic treatment were carried into the growth phase for the next evolution round, in order to generate a similar bottleneck as mitomycin C and gentamicin treatment. Shown is the mean of three biological replicates; error bars denote SEM. Source data are provided as a Source Data file.

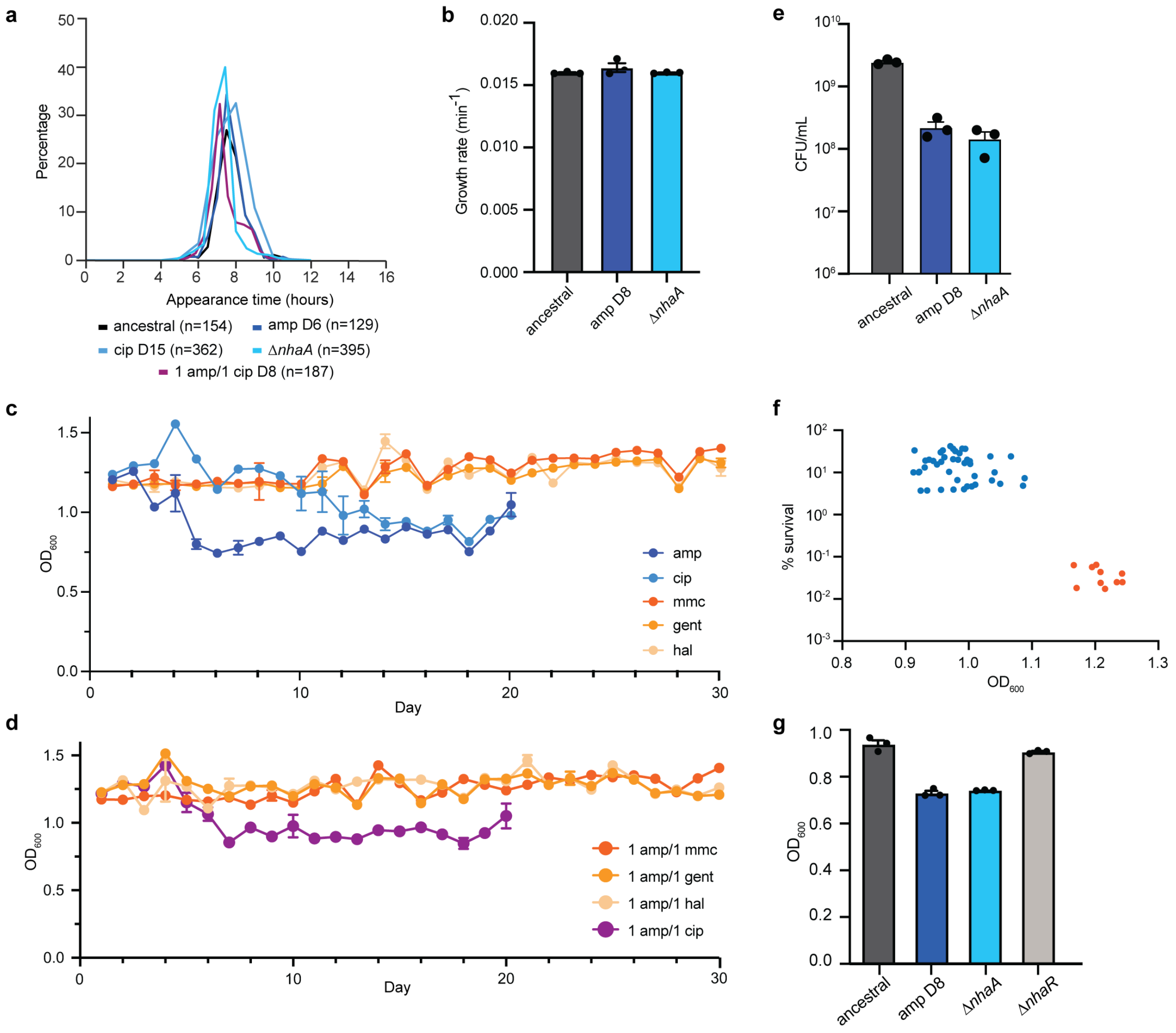

Supplementary Figure 4. Tolerance acquisition is linked to reduced stationary-phase density, not a change in lag time or growth rate. Related to Fig. 2. **a,** Colony appearance time analysis. Cells were spread on LB agar plates and scans were taken every 5 minutes. Time-lapse images were analyzed using ColTapp[19]. The number of colonies analyzed under each condition is indicated in the legend. **b,** Growth rate determination. The log phase of growth curves conducted in LB broth was fit to an exponential growth equation and the growth rate was extracted. Shown is the mean of three biological replicates; error bars represent SEM. **c-d,** Daily optical density

measurements of the overnight cultures from monotherapy (single antibiotic) evolutions (c) or evolutions where two antibiotics were cycled every day (d). Evolutions were conducted in biological triplicate; error bars denote SEM. **e,** Cell counts from overnight, stationary-phase cultures. Data are representative of three biological replicates; error bars indicate SEM. **f,** Clonal analysis of amp D8. 53 clones were isolated, and their stationary-phase optical density and survival against ampicillin were measured. Blue data points indicate tolerant clones that have a reduced stationary-phase density, orange data points indicate non-tolerant clones that do not have a reduced stationary-phase density. **g,** Stationary-phase optical density measurements of ancestral, amp D8, *ΔnhaA,* and *ΔnhaR* in LB broth. Data are representative of three biological replicates; error bars indicate SEM. Source data are provided as a Source Data file.

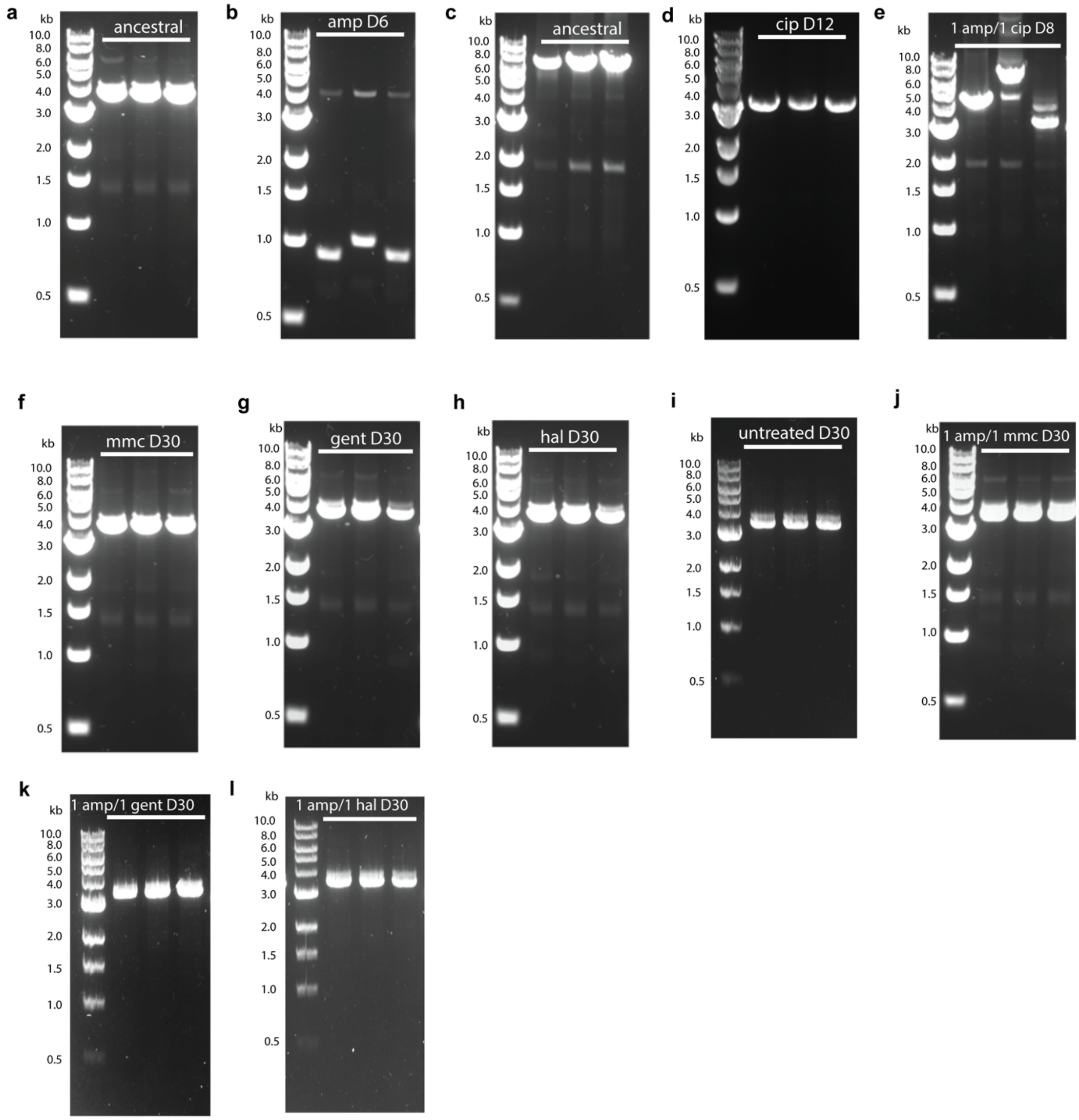

Supplementary Figure 5. Agarose gel images showing colony PCR products from the indicated evolutions. Related to Fig. 2. Three colony PCR reactions were performed for each condition, with replicates 1-3 in order from left to right. Panels C-E were amplified with PCR primer set 2, while all others were amplified with primer set 1. Ladder sizes are displayed on the left lane of each gel. PCR products with a deletion were selected for Sanger sequencing as shown in Supplementary Table 4. Source data are provided as a Source Data file.

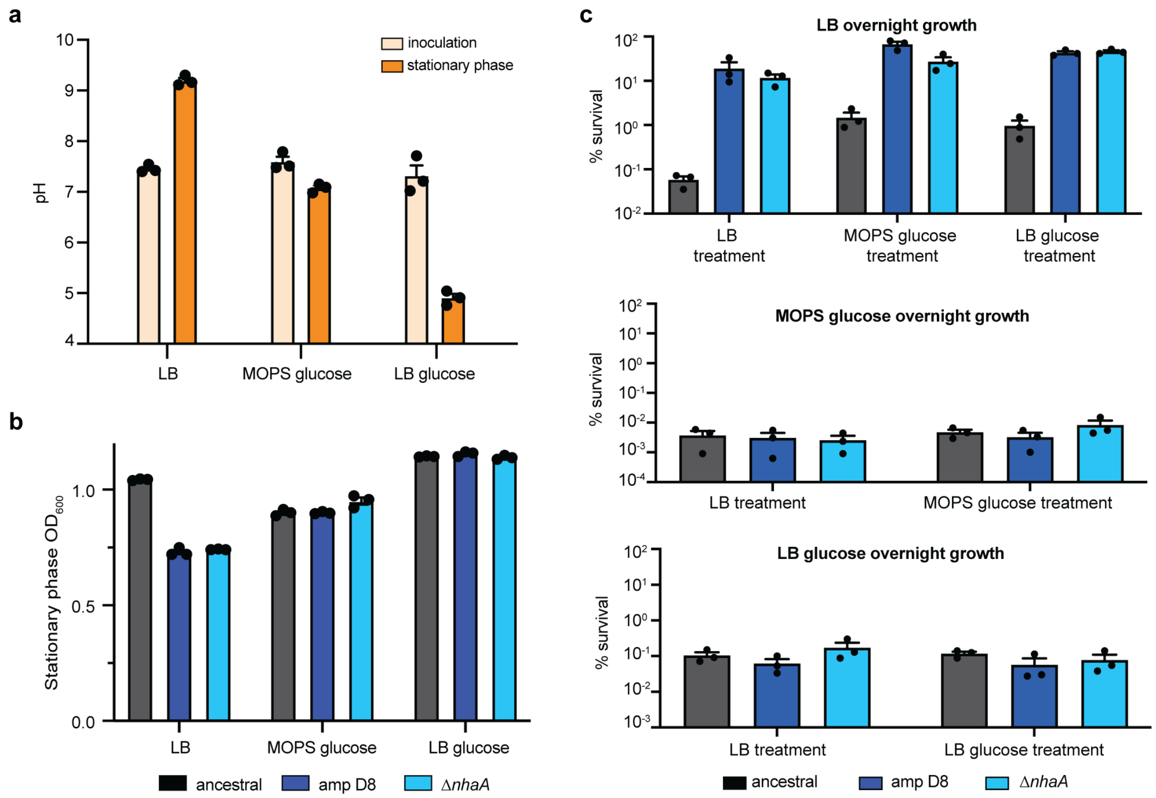

Supplementary Figure 6. Reduced density and tolerance in SDM-evolved cells are dependent on media alkalization. Related to Fig. 2. **a,** Extracellular pH measurements of cultures grown in LB, MOPS 0.2% glucose, and LB 0.2% glucose, at the time of inoculation and at stationary phase. Data are representative of three biological replicates; error bars show SEM. **b,** Stationary-phase optical density of cultures grown in LB, MOPS 0.2% glucose, or LB 0.2% glucose. Three biological replicates are shown; error bars represent SEM. **c,** Dependence of the tolerance phenotype on the overnight growth media. Cells were cultured overnight in LB (top), MOPS glucose (middle), or LB glucose (bottom), then washed and diluted 1:100 in fresh LB, MOPS glucose, or LB glucose and treated with ampicillin. Figure titles indicate the media used for overnight growth and horizontal axis labels indicate the media used during ampicillin treatment. Survival assays were conducted in biological triplicate; error bars denote SEM. Source data are provided as a Source Data file.

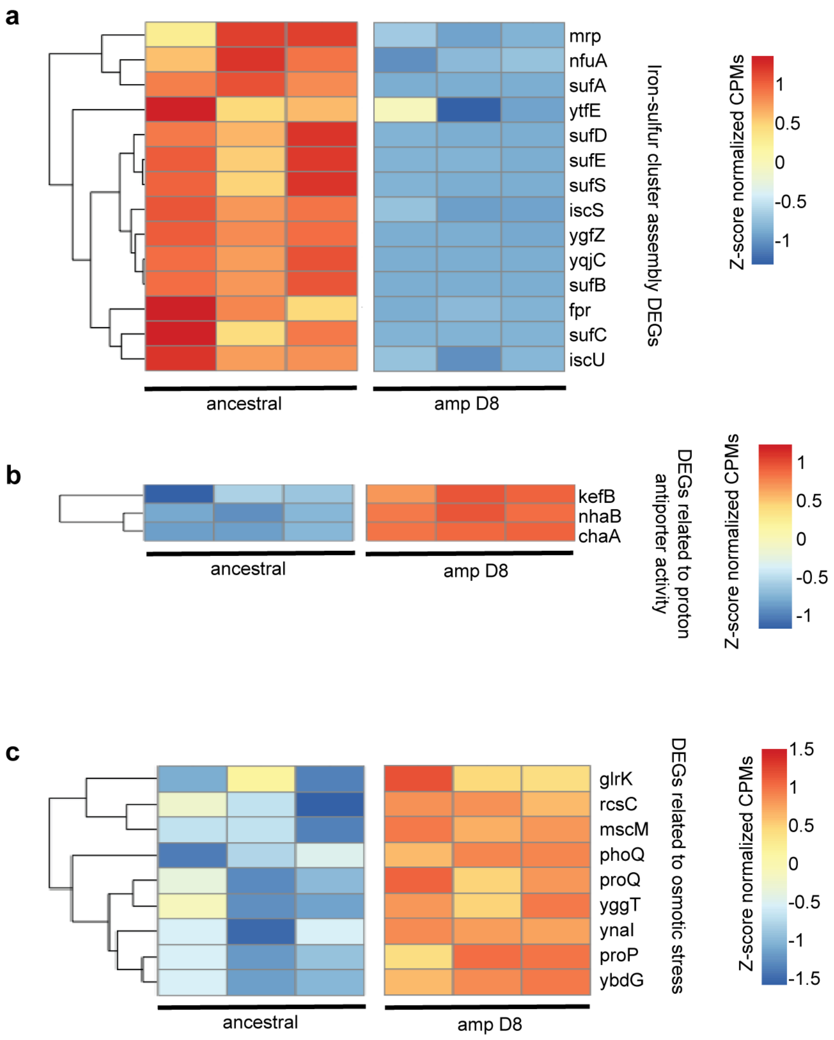

Supplementary Figure 7. Additional RNA-seq heatmaps. Related to Fig. 2. Additional heatmaps for iron-sulfur cluster assembly (GO:0016226) (a), as well as transcripts associated with proton antiporter activity (b) and osmotic stress (c). Heatmaps illustrate the hierarchical clustering of z-score normalized counts per million. Source data are provided as a Source Data file.

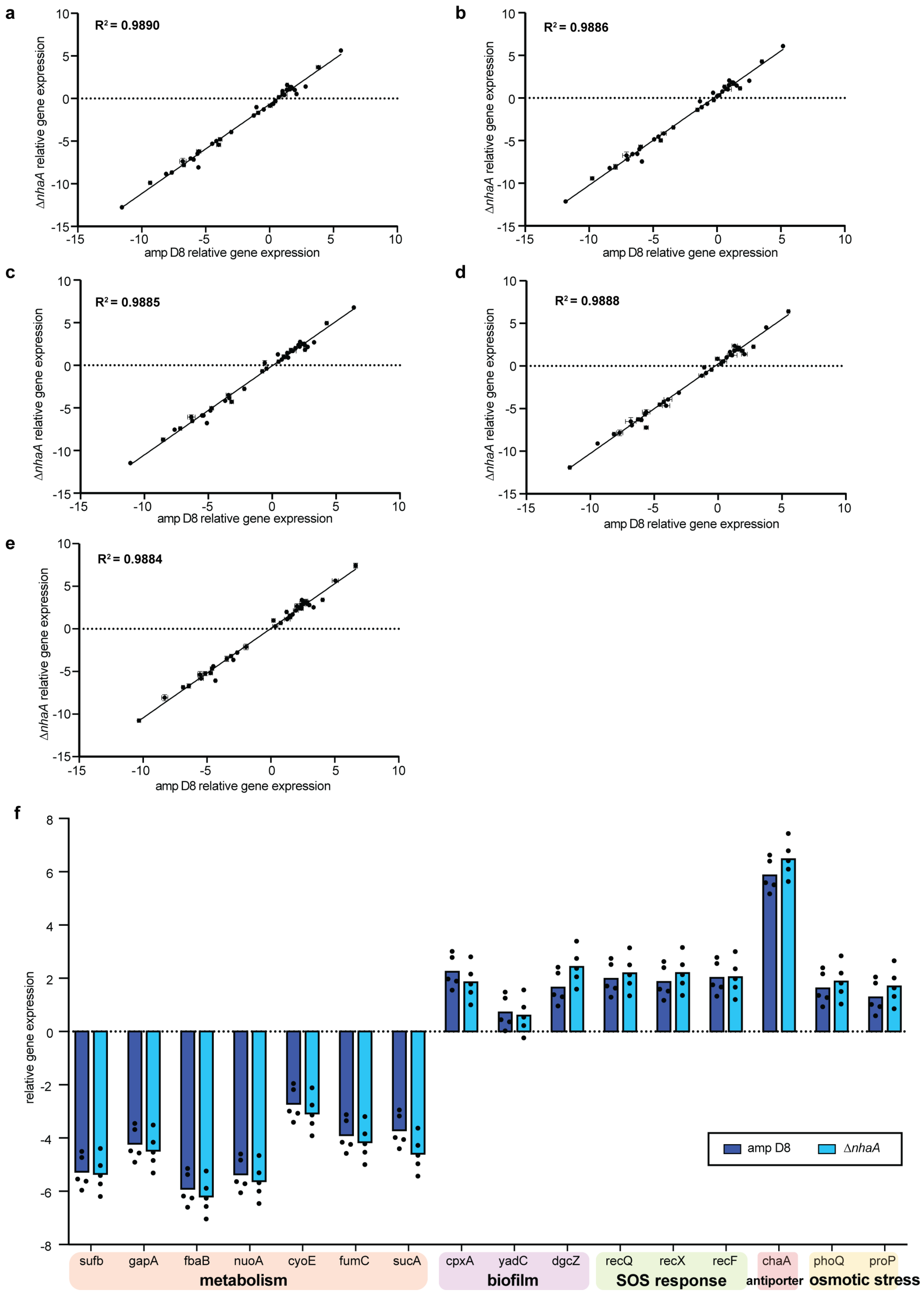

a
R² = 0.9890
ΔnhaA relative gene expression
amp D8 relative gene expression
b
R² = 0.9886
c
R² = 0.9885
d
R² = 0.9888
e
R² = 0.9884
f
relative gene expression
amp D8
ΔnhaA
sufb
gapA
fbaB
nuoA
cyoE
fumC
sucA
cpxA
yadC
dgcZ
recQ
recX
recF
chaA
phoQ
proP
metabolism
biofilm
SOS response
antiporter
osmotic stress

Supplementary Figure 8. Amp D8 and Δ*nhaA* have similar gene expression profiles. Related to Fig. 2. **a-e,** High correlation of relative gene expression between amp D8 and Δ*nhaA*. qRT-PCR was performed on 40 transcripts from ancestral, amp D8, and Δ*nhaA*. Threshold cycle (Ct) values from the ancestral strain were used as the control. Relative gene expression (i.e., negative ΔΔCt) was calculated using five different housekeeping genes determined as non-differentially expressed in the RNA-sequencing analysis between ancestral and amp D8. Housekeeping genes used were epmC (a), fliF (b), tsaD (c), glpT (d), and argH (e). qRT-PCR reactions were performed in biological duplicate and technical triplicate, and error bars represent SEM between biological replicates. **f,** Relative gene expression of specific transcripts related to metabolism, biofilm formation, SOS response, antiporter activity, and osmotic stress. Bars represent the mean of negative ΔΔCt values calculated using five different housekeeping genes. Each data point is the mean of two biological replicates. Source data are provided as a Source Data file.

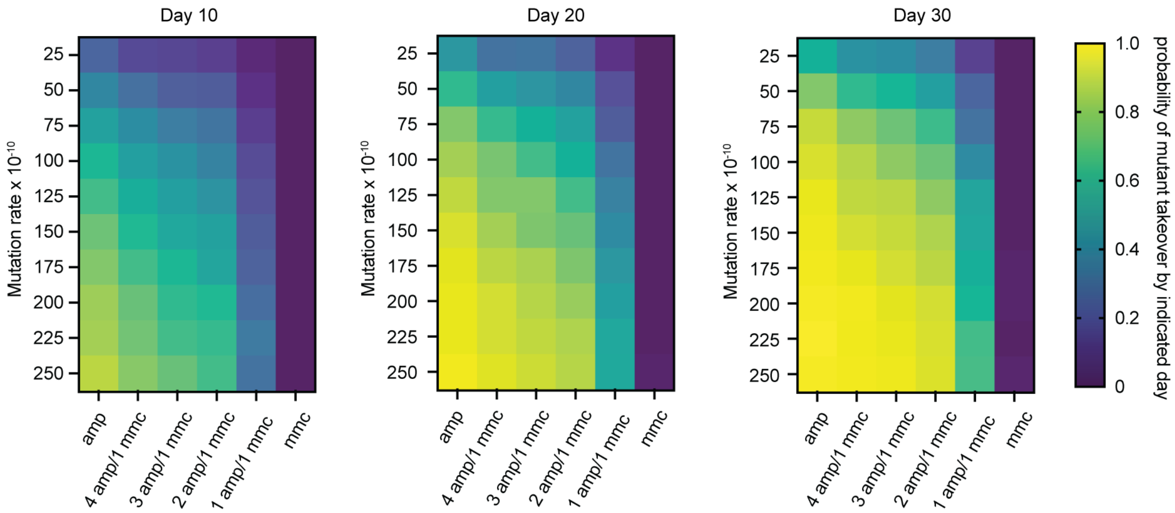

Supplementary Figure 9. Sensitivity of the model to mutation rate. Related to Fig. 3. The model was run with a range of mutation rates and the probability of mutant takeover (defined by when the number of tolerant mutant cells exceeds the number of wild-type cells) by the indicated day was calculated using the results from 1000 simulations for each cycling regimen. Source data are provided as a Source Data file.

Designation	Description	Genotype	Source
ancestral	*E. coli* strain BW25113	F - , Δ(araD-araB)567, ΔlacZ4787(::rrnB-3), λ - , rph-1, Δ(rhaD-rhaB)568, hsdR514	Lab stock
ΔnhaA	*E. coli* strain BW25113 with *nhaA* knocked out and replaced with kanamycin resistance	F -, Δ*nhaA737::kan*, Δ(araD-araB)567, ΔlacZ4787(::rrnB-3), λ - , rph-1, Δ(rhaD-rhaB)568, hsdR514	Keio collection; JW0018
ΔnhaR	*E. coli* strain BW25113 with *nhaR* knocked out and replaced with kanamycin resistance	F - , Δ(*nhaR738::kan,* Δ(araD-araB)567, ΔlacZ4787(::rrnB-3), λ - , rph-1, Δ(rhaD-rhaB)568, hsdR514	Keio collection; JW0019

Supplementary Table 1. Strains used in this study.

	150 µg/mL	120 µg/mL	60 µg/mL	30 µg/mL	15 µg/mL
slope	-0.09652	-0.09652	-0.06845	0.8867	0.7740
R^2	0.2059	0.2059	0.003735	0.2850	0.4320

Supplementary Table 2. Slope and R^2 for linear regressions at various halicin concentrations from Supplementary Fig. 1.

Genomic position	Mutation in Amp D8 Replicate 1	Mutation in Amp D8 Replicate 2	Notes
17115	2680 bp deletion		Deletion of *nhaA* and *nhaR*
17242		2553 bp deletion	Deletion of *nhaA* and *nhaR*
20569	1 bp insertion		Between IS1A and 30S ribosomal protein

Supplementary Table 3. List of variants identified in whole-genome sequencing. Long variants labeled as “imprecise” by Pilon (containing N’s in the local reassembly) were filtered out.

Evolution condition	Gel figure	Replicate	PCR primer set	Deletion coordinates	Deletion size (kb)
Ancestral	A	1-3	1	none	-
	C		2		
Amp Day 6	B	1	1	17115-19795	2.680
		2		17242-19795	2.553
		3		17139-19795	2.656
Cip Day 12	D	1	2	15857-19795	3.938
		2		15857-19795	3.938
		3		15857-19795	3.938
Amp/cip Day 8	E	1	2	17075-19795	2.720
		2		none	-
		3		15896-19795	3.899
Mmc Day 30	F	1-3	1	none	-
Gent Day 30	G	1-3	1	none	-
Hal Day 30	H	1-3	1	none	-
Untreated Day 30	I	1-3	1	none	-
1 day amp/1 day mmc Day 30	J	1-3	1	none	-
1 day amp/1 day gent Day 30	K	1-3	1	none	-
1 day amp/1 day hal Day 30	L	1-3	1	none	-

Supplementary Table 4. Summary of deletion events identified in colony PCR and Sanger sequencing, related to Supplementary Fig. 5.

Parameter	Value	Description
Wild-type stationary phase density (CFU/mL)	[2.29×10^9 – 2.71×10^9]	From experimental data
Mutant stationary phase density (CFU/mL)	[1.57×10^8 – 3.14×10^8]	From experimental data
SDM wild-type survival (% survival)	[.02 - .0667]	From experimental data with ampicillin treatment
SDM mutant survival (% survival)	[5.56 – 55.2]	From experimental data with ampicillin treatment
WDM wild-type survival (% survival)	[.00226 - .00548]	From experimental data with mitomycin C treatment
WDM mutant survival (% survival)	[.005 - .0171]	From experimental data with mitomycin C treatment
Culture volume (mL)	0.4	Consistent with experimental protocol
Fold dilution before treatment	100	Consistent with experimental protocol
Mutation rate (per division)	50×10^{-10}	From literature[20]. Used unless otherwise noted.

Supplementary Table 5. Model parameters.

References

1. Dover, N. & Padan, E. Transcription of nhaA, the Main Na^+/H^+ Antiporter of *Escherichia coli*, Is Regulated by Na^+ and Growth Phase. *J. Bacteriol.* **183**, 644-653 (2001).
2. Padan, E. Functional and structural dynamics of NhaA, a prototype for Na^+ and H^+ antiporters, which are responsible for Na^+ and H^+ homeostasis in cells. *Biochim. Biophys. Acta.* **1837**, 1047-1062 (2014).
3. Bourque, G., *et al*. Ten things you should know about transposable elements. *Genome Biol*. **19**, 199 (2018).
4. Dwyer, D.J. et al. Antibiotics induce redox-related physiological alterations as part of their lethality. *Proc. Natl. Acad. Sci. U.S.A.* **111**, E2100-E2109 (2014).
5. Hong, S.H., Wang, X., O'Connor, H.F., Benedik, M.J. & Wood, T.K. Bacterial persistence increases as environmental fitness decreases. *Microb. Biotech.* **5**, 509-522 (2012).
6. Imlay, J. A. The molecular mechanisms and physiological consequences of oxidative stress: lessons from a model bacterium. *Nat. Rev. Micro.* **11**, 443-454 (2013).
7. Rowe, S. E. *et al.* Reactive oxygen species induce antibiotic tolerance during systemic *Staphylococcus aureus* infection. *Nat. Microbiol.* **5**, 282-290 (2020).
8. Kohanski, M. A., Dwyer, D. J., Hayete, B., Lawrence, C. A. & Collins, J. J. A Common Mechanism of Cellular Death Induced by Bactericidal Antibiotics. *Cell* **130**, 797–810 (2007).
9. Padan, E., Bibi, E., Ito, M. & Krulwich, T.A. Alkaline pH homeostasis in bacteria: New insights. *Biochim. Biophys. Acta.* **1717**, 67-88 (2005).

10. Schuldiner, S. et al. Induction of SOS Functions by Alkaline Intracellular pH. *J. Bacteriol.* **168**, 936-939 (1986).
11. Podlesek, Z. & Bertok, D. Ž. The DNA Damage Inducible SOS Response Is a Key Player in the Generation of Bacterial Persister Cells and Population Wide Tolerance. *Front. Microbiol.* **11**, 1785 (2020).
12. Dörr, T., Lewis, K. & Vulic, M. SOS Response Induces Persistence to Fluoroquinolones in *Escherichia coli*. *Plos. Genet.* **5**, e1000760 (2009).
13. Prax, M. & Bertram, R. Metabolic aspects of bacterial persisters. *Front. Cell. Infect. Microbol.* **4**, 148 (2014).
14. Wood, T. K., Knabel, S. J. & Kwan, B. W. Bacterial Persister Cell Formation and Dormancy. *Appl. Environ. Microbio*l. **79**, 7116-7121 (2013).
15. Ohyama, T., Igarashi, I. & Kobayashi, H. Physiological Role of the *chaA* Gene in Sodium and Calcium Circulations at a High pH in *Escherichia coli*. *J. Bacteriol.* **176**, 4311-4315 (1994).
16. Krulwich, T. A., Sachs, G. & Padan, E. Molecular aspects of bacterial pH sensing and homeostasis. *Nat. Rev. Micro.* **9**, 330-343 (2011).
17. Lopatkin, A. J. *et al.* Bacterial metabolic state more accurately predicts antibiotic lethality than growth rate. *Nat. Microbiol.* **19**, 1–9 (2019).
18. Zheng, E. J., Stokes, J. M. & Collins, J. J. Eradicating Bacterial Persisters with Combinations of Strongly and Weakly Metabolism-Dependent Antibiotics. *Cell Chem. Biol.* **27**, 1–9 (2020).
19. Bär, J., Boumasmoud, M., Kouyos, R. D., Zinkernagel, A. S. & Vulin, C. Efficient microbial colony growth dynamics quantification with ColTapp, an automated image analysis application. *Sci. Rep.* **10**, 16084 (2020).
20. Levin-Reisman, I. et al. Antibiotic tolerance facilitates the evolution of resistance. *Science* **355**, 827-830 (2017).
