# Supplementary material for: Modulating the evolutionary trajectory of tolerance using antibiotics with different metabolic dependencies
Source: Nat Commun. 2022 May 9;13:2525. doi: 10.1038/s41467-022-30272-0 (PMC9085803; doi:10.1038/s41467-022-30272-0)
Supplement: Supplementary file 2 — Reporting Summary [file 41467_2022_30272_MOESM2_ESM.pdf]

nature portfolio

Corresponding author(s): James J. Collins

Last updated by author(s): April 10, 2022

Reporting Summary

Nature Portfolio wishes to improve the reproducibility of the work that we publish. This form provides structure for consistency and transparency in reporting. For further information on Nature Portfolio policies, see our Editorial Policies and the Editorial Policy Checklist.

Statistics

For all statistical analyses, confirm that the following items are present in the figure legend, table legend, main text, or Methods section.

n/a	Confirmed	
☐	☒	The exact sample size (n) for each experimental group/condition, given as a discrete number and unit of measurement
☐	☒	A statement on whether measurements were taken from distinct samples or whether the same sample was measured repeatedly
☐	☒	The statistical test(s) used AND whether they are one- or two-sided<br>*Only common tests should be described solely by name; describe more complex techniques in the Methods section.*
☒	☐	A description of all covariates tested
☐	☒	A description of any assumptions or corrections, such as tests of normality and adjustment for multiple comparisons
☐	☒	A full description of the statistical parameters including central tendency (e.g. means) or other basic estimates (e.g. regression coefficient) AND variation (e.g. standard deviation) or associated estimates of uncertainty (e.g. confidence intervals)
☐	☒	For null hypothesis testing, the test statistic (e.g. F, t, r) with confidence intervals, effect sizes, degrees of freedom and P value noted<br>*Give P values as exact values whenever suitable.*
☒	☐	For Bayesian analysis, information on the choice of priors and Markov chain Monte Carlo settings
☒	☐	For hierarchical and complex designs, identification of the appropriate level for tests and full reporting of outcomes
☒	☐	Estimates of effect sizes (e.g. Cohen's d, Pearson's r), indicating how they were calculated

Our web collection on statistics for biologists contains articles on many of the points above.

Software and code

Policy information about availability of computer code

Data collection	Code for the SDM/WDM model is available as Supplementary Software.
Data analysis	Graphpad Prism 8 was used for regression analysis. BWA version v0.7.17 and Pilon version v1.23 were used for analysis of DNA sequencing data. FastQC (11.9), Bowtie2 (2.3.4.3), HTSeq (2.0.1), EdgeR (version 3.32.1), and OmicsBox (2.0.36) were used for RNA seq analysis. MATLAB version R2021a was used for model building. ColTapp was used for colony appearance time analysis of time-lapsed images. LightCycler Software (Roche) was used to determine threshold cycle (Ct) values for qRT-PCR.

For manuscripts utilizing custom algorithms or software that are central to the research but not yet described in published literature, software must be made available to editors and reviewers. We strongly encourage code deposition in a community repository (e.g. GitHub). See the Nature Portfolio guidelines for submitting code & software for further information.

Data

Policy information about availability of data

All manuscripts must include a data availability statement. This statement should provide the following information, where applicable:

- Accession codes, unique identifiers, or web links for publicly available datasets
- A description of any restrictions on data availability
- For clinical datasets or third party data, please ensure that the statement adheres to our policy

The sequencing data generated in this study have been deposited in the Sequence Read Archive repository under accession code PRJNA810430 [https://www.ncbi.nlm.nih.gov/bioproject/PRJNA810430]. The reference E. coli BW25113 genome used in this study is available in the NCBI database under accession code CP009273.1 [https://www.ncbi.nlm.nih.gov/nuccore/CP009273]. Source data are provided with this paper.

Field-specific reporting

Please select the one below that is the best fit for your research. If you are not sure, read the appropriate sections before making your selection.

- [x] Life sciences
- [ ] Behavioural & social sciences
- [ ] Ecological, evolutionary & environmental sciences

For a reference copy of the document with all sections, see nature.com/documents/nr-reporting-summary-flat.pdf

Life sciences study design

All studies must disclose on these points even when the disclosure is negative.

Sample size	Sample sizes for all experiments were chosen as per field convention (PMID 25043002, PMID 32916087, PMID 31920087). Bacterial survival assays and MIC experiments were performed in biological duplicate or triplicate as indicated in the figure legend. qRT-PCR experiments were performed in technical triplicate and biological duplicate. DNA and RNA sequencing was conducted in biological duplicate or triplicate as indicated.
Data exclusions	No data was excluded from analysis.
Replication	Data is representative of at least two biological replicates, with the number of replicates for each experiment indicated in the figure legends. All attempts at replication were successful.
Randomization	Since each experiment was conducted by a single person, randomization was not possible.
Blinding	Since each experiment was conducted by a single person, and sample labeling was required, blinding was not possible. Additionally, blinding was not required because all experiments were performed through quantitative techniques.

Reporting for specific materials, systems and methods

We require information from authors about some types of materials, experimental systems and methods used in many studies. Here, indicate whether each material, system or method listed is relevant to your study. If you are not sure if a list item applies to your research, read the appropriate section before selecting a response.

Materials & experimental systems

n/a	Involved in the study	
[x]	[ ]	Antibodies
[x]	[ ]	Eukaryotic cell lines
[x]	[ ]	Palaeontology and archaeology
[x]	[ ]	Animals and other organisms
[x]	[ ]	Human research participants
[x]	[ ]	Clinical data
[x]	[ ]	Dual use research of concern

Methods

n/a	Involved in the study	
[x]	[ ]	ChIP-seq
[x]	[ ]	Flow cytometry
[x]	[ ]	MRI-based neuroimaging
