# Supplementary material for: Modulating the evolutionary trajectory of tolerance using antibiotics with different metabolic dependencies
Source: Nat Commun. 2022 May 9;13:2525. doi: 10.1038/s41467-022-30272-0 (PMC9085803; doi:10.1038/s41467-022-30272-0)
Supplement: Supplementary file 3 — Description of Additional Supplementary Files [file 41467_2022_30272_MOESM3_ESM.pdf]

Description of Additional Supplementary Files

Title: Supplementary Data 1:

Description: Primers used in this study.

Title: Supplementary Data 2:

Description: RNA sequencing data.

Sheet 1 Upregulated Genes. Sheet 2 Downregulated Genes. Genes were determined as up or downregulated using the QL F-test in EdgeR, with standard cutoffs (a two-sided p-value cut-off of <0.05) and corrected for multiple comparisons using FDR <0.05 with the Benjamini-Hochberg method.

Sheet 3 Fisher's exact test: Upregulated Enriched GOs. Sheet 4 Fisher's exact test: Downregulated Enriched GOs. Enriched GO terms were determined using Fisher's exact test. Exact P-values are listed, corrected for multiple comparisons using FDR <0.05 with the Benjamini-Hochberg method.

Sheet 5 GSEA: Upregulated Enriched GOs. Sheet 6 GSEA: Downregulated Enriched GOs. GO terms were determined as enriched using GSEA (Gene set enrichment analysis), NES normalized enrichment scores are listed. Exact *P*-values are listed, corrected for multiple comparisons using FDR method.

Sheet 7 Gene IDs and GOs. All gene IDs and their annotated GO terms. While not listed, parent GO terms are included in all analyses according to the "true path" rule.

Title: Supplementary Software:

Description: Code for the SDM/WDM cycling prediction model.
